# First systematic inventory of the jumping plant lice of Luxembourg (Hemiptera, Sternorrhyncha, Psylloidea)

**DOI:** 10.3897/BDJ.10.e77571

**Published:** 2022-08-04

**Authors:** Carmelo Rapisarda, Alexander M. Weigand, Paul Braun, Michael Eickermann

**Affiliations:** 1 Dipartimento di Agricoltura, Alimentazione e Ambiente (Di3A), Università degli Studi di Catania, Catania, Italy Dipartimento di Agricoltura, Alimentazione e Ambiente (Di3A), Università degli Studi di Catania Catania Italy; 2 Musée National d'Histoire Naturelle (MNHNL), Luxembourg, Luxembourg Musée National d'Histoire Naturelle (MNHNL) Luxembourg Luxembourg; 3 Luxembourg Institute of Science and Technology, Belvaux, Luxembourg Luxembourg Institute of Science and Technology Belvaux Luxembourg

**Keywords:** Psyllids, Luxembourg, species diversity, biology, ecology

## Abstract

Psyllids (superfamily Psylloidea), also known as jumping plant lice, are a group of plant-sap sucking Hemiptera having significant pest status for crops, forest trees and ornamental plants. Only seven species of psyllids have been recorded in Luxembourg so far. An additional group of seven species has been recorded exclusively, based on the findings of their galls or specific plant deformations; but no mention exists in literature on the actual collection of the inducing insect in Luxembourg. To fill this knowledge gap, field collections were carried out during the years 2019-2020. In addition, samples from 1999-2000 stored in the wet collection of the Musée National d’Histoire Naturelle de Luxembourg were studied. This research, in combination with information coming from literature, allowed us to list 48 species of the families Aphalaridae (5 species), Liviidae (5), Psyllidae (24) and Triozidae (14), though the presence of one species within the last family (*Triozarhamni*) needs to be confirmed. Brief information on geographical distribution, biology and (if available) illustrations of diagnostic characters are provided on the psyllid species detected in Luxembourg so far.

## Introduction

Psyllids (superfamily Psylloidea), also known as jumping plant lice, are a group of plant-sap sucking Hemiptera which may have significant pest status for crops, forest trees and ornamental plants due to their copious production of honeydew, their frequent coating with waxy secretions (smearing the canopy of infested plants), the injection of toxic saliva (causing necrosis, deformations or galls) and, last but not least, their responsibility in transmitting many pathogens to plants, mainly bacteria and especially phytoplasmas (Hodkinson 1974, Hodkinson 1984, Burckhardt 1994a, Burckhardt 2005, Munyaneza 2010, Haapalainen 2014, Ben Othmen et al. 2019). In spite of their importance, psyllids are still poorly known in Luxembourg; this is why their species composition, diversity and distribution need to be properly assessed.

According to literature, *Spanioneurabuxi* (Linnaeus), *S.fonscolombii* Foerster and *Trichochermeswalkeri* (Foerster) are the only species of Psylloidea whose presence in Luxembourg has been reported till now based on adult specimens (Baugnée 2001, O’Connor and Malumphy 2011, Eickermann et al. 2020). However, additional records of psyllids can be also considered, deriving from rich cecidological literature dealing with the presence of plants with galls or deformations probably induced by Psylloidea. Yet, these records need to be confirmed by finding the insects to which the effects on plants are attributed. In particular, Lambinon and Schneider (2004) investigated deformations caused to plants by *Cacopsyllamali* (Schmidberger), *C.melanoneura* (Foerster), *Liviajunci* (Schrank), *Spanioneurabuxi* (Linnaeus), *Psyllopsisfraxini* (Linnaeus), *Lauritriozaalacris* (Flor), *Triozaflavipennis* Foerster, *T.remota* Foerster and *T.rhamni* (Schrank), in addition to the ones caused by *T.walkeri* (already reported for Luxembourg). For *P.fraxini*, the authors reported the rear of the psyllid from leaf galls collected on *Fraxinusexcelsior* L. from many localities in Luxembourg (Bettembourg, Bonnevoie, Bridel and Kleinbettingen). Similarly, they reported findings of eggs of *T.flavipennis* in pit galls found on leaves of *Aegopodiumpodagraria* L. For all remaining cecidia described, however, no indication was given on the finding of the causing insect. Deformations caused by three additional psyllid species [*Camarotoscenaspeciosa* (Flor), *Triozacentranthi* (Vallot) and *T.urticae* (Linnaeus)] were reported by Lambinon et al. (2012), who did not mention any finding of their causing insects. Deformations by *L.alacris* and *T.centranthi* and galls by *T.remota* were reported again by Schneider (2016) from new localities in Luxembourg. Only for *T.remota*, the author referred to the presence of an insect immature within each pit gall found on leaves of *Quercuspetraea* (Matt.) Liebl. and *Q.robur* L. Cecidia caused by *C.speciosa*, *L.junci* and *T.flavipennis* were reported again by Burton et al. (2019), who explicitly mentioned for the first time the finding of nymphs of *C.speciosa* and their abundant wax secretion on the leaf deformations of *Populusnigra* L. and confirmed the occurrence of a *T.flavipennis* nymph in the concave leaf gall produced on *A.podagraria*.

Considering the available literature data, seven psyllid species were known for Luxembourg, based on observations of specimens (*Camarotoscenaspeciosa*, *Psyllopsisfraxini*, *Spanioneurabuxi*, *S.fonscolombii*, *Trichochermeswalkeri*, *Triozaflavipennis* and *T.remota*) and seven further species were only recorded, based on the findings of galls or deformations they cause to plants (*Cacopsyllamali*, *C.melanoneura*, *Liviajunci*, *Lauritriozaalacris*, *Triozacentranthi*, *T.rhamni* and *T.urticae*).

Even when hypothesising that all psyllid records, based on findings of their galls, will be confirmed by collection of insect specimens, still the number of psyllid species presently known in Luxembourg is remarkably low, for example, when compared with faunas of neighbouring countries in the “Benelux” region”: 69 species known in The Netherlands (den Bieman et al. 2019); at least 56 species in Belgium (Ouvrard 2021). Present knowledge on the psyllid fauna of Luxembourg appears weak also when considering the richness and composition of the regional flora (especially for plant groups that may host psyllid species), as assessed by Lambinon and Verloove (2012). The lack of a systematic inventory of the psyllids of Luxembourg, in combination with their ecological and partly agricultural significance, gave rise to the current manuscript.

## Data resources

### Study area

The Grand Duchy of Luxembourg is characterised by a temperate, semi-oceanic climate. Even though the area of the country is small (2,586 km^2^) with a maximal Euclidean distance of 82 km from north to south and 57 km from east to west, Luxembourg offers quite diverse physiogeography with different climatological characteristics, associated vegetation and anthropogenic land use. Traditionally, Luxembourg is divided into two main ecoregions, the Oesling (32% of total area) and the Gutland (68%) (Dohet et al. 2008). The Oesling is located in the north of the country, in the border region between Belgium and Luxembourg – and is the eastern part of woody mountains called the Ardennes with highest altitude of 450 m a.s.l. Annual precipitation ranges from 800–1000 mm and annual mean air temperature is 7.5°C (Goergen et al. 2013). The region is characterised by meadows, pastures and forests of coniferous/deciduous trees. The Gutland shows a higher level of anthropogenic disturbance (including larger cities and industrial areas) and a longer vegetational period in comparison to the Oesling (Goergen et al. 2013). Annual precipitation ranges from 700–800 mm and annual mean air temperature is 9°C (Goergen et al. 2013). It can be divided into four sub-ecoregions: the Western and the Eastern Gutland, the Moselle Valley and the Minette Basin. Land use is quite diverse in the Gutland. Pastures are still common, while the acreage of arable land is increasing. Extended deciduous forests are typical for the Gutland (Dietz and Pir 2009). For the Western Gutland, secondary sandstones and sandy soils are common (Stevens et al. 2010) and the amount of precipitation is higher compared to the eastern part (Goergen et al. 2013). The Eastern Gutland is characterised by loam-loess-based soils and a more variable topography (Stevens et al. 2010). The Minette Basin is located in the south-western part of Luxembourg, next to the French border. It represents the former mining district, due to the rich resources of iron ore. The Moselle Valley – along the border to Germany – represents the smallest ecoregion (1% of total area of Luxembourg). It is sunnier and also drier (less than 700 mm) in comparison to all other areas of the country. Due to these very specific climatic conditions, vineyards are prevalent and agriculture shows a higher level of intensification (Dohet et al. 2008). For further information about climatological characteristics of the different regions in Luxembourg, see Eickermann et al. (2014).

### Field collection of psyllids, their preparation and identification (2019/2020)

Adult psyllids were collected during one year (July 2019 to June 2020) by beating host or shelter plants over a sweeping-net, from which the specimens were captured into plastic tubes containing 70% ethanol, thereby exploiting their natural aptitude to jump. When easily visible on the plants, nymphs were also collected by picking them directly and storing them in plastic tubes as described for the adults. If necessary, plants on which samples were collected (or parts of them) were stored in plastic bags and taken to the laboratory for their specific identification, according to Lambinon and Verloove (2012).

In the lab, all specimens collected in each tube were observed using a dissecting microscope (LEICA MZ7.5); in this phase, adults were separated by sex, counted and (where possible) identified. For samples whose observation under a dissecting microscope allowed the species identification, all specimens (adults and nymphs, if present) were stored in glass tubes containing 70% ethanol, each marked with a progressive collection number, corresponding to those of the general collection register containing complete data on the date and location of collection (including the geographical coordinates) and on the plant(s) on which the sample was recorded. In case a closer examination was needed to allow species identification, a maximum of six specimens (if available, three of each sex) were mounted on permanent microscope slides. For slide preparation, selected adults were firstly cleaned in 70% ethanol under gentle heating, then left to clear overnight in 10% potassium hydroxide (KOH), rinsed in a solution of 20% glacial acetic acid, dehydrated in 95% ethanol (for 10 minutes), placed in xylene for ≥ 10 minutes, mounted in Canada Balsam and allowed to dry in an incubator for 15 days at 35°C.

Each specimen was dissected in a drop of mounting medium before being mounted on the slide, in order to mount all different parts of the body separately: head (mounted dorsal uppermost), pronotum and forelegs, mouth parts, mesoscutum and forewings (dorsally), ventral mesothorax and mid-legs, metathorax, hind legs and abdomen (laterally, with well-exposed genitalia).

Photographs of morphological details (head, forewing, male and female terminalia) were made at the Musée National d’Histoire Naturelle de Luxembourg (MNHNL), from ethanol-preserved specimens of most of the species collected, using a Keyence VHX-6000 digital microscope.

With the exception of some special cases, for which we used literature specifically concerning the taxonomic group of the species to be identified, the material was identified by using the taxonomic keys of Hodkinson and White (1979) and Ossiannilsson (1992), following the classification and nomenclature proposed by Burckhardt et al. (2021).

All the material studied, i.e. wet and slide mounted material, is stored at the MNHNL, apart from several specimens being part of abundant field collected samples, which are stored in the collections of the museum of the University of Catania (Italy).

### Study of psyllid material from Malaise and Moericke traps (1999/2000)

During the years 1999-2000, an entomological collection campaign was carried out in Luxembourg by Evelyne Carrières, focussing on the national inventory of hoverflies (Syrphidae) (Carrières 2003). The project was financed by the MNHNL. Several Malaise and yellow Moericke traps were deployed in various areas of the country and regularly controlled every 2-4 weeks after initial installation. Many of the bulk samples collected during this campaign (and stored in the MNHNL wet collection) contained psyllid specimens, which, as part of the research aimed at compiling this manuscript, were sorted out, observed under a LEICA dissecting microscope, separated by sex, counted and (where possible) identified as already reported in the previous section. Additionally, in this case, all material identified by the simple use of a dissecting microscope has been stored in glass tubes containing 70% ethanol and marked with progressive collection numbers (different from those used for the field collected material). A general register has been prepared, containing complete data on collection localities (including the geographical coordinates) and the dates of exposure and removal of the traps. In case a more sophisticated approach was necessary to identify some material, permanent slides were prepared and identifications performed as described in the previous section. All material studied, both ethanol preserved or mounted on permanent slides, is stored at the MNHNL.

## Results

Overall, the reliable bibliographic references (e.g. those reporting the actual collection of Psylloidea specimens), the field collections we made during 2019/2020 and the investigated Malaise trap material from 1999/2000 stored at the MNHNL allowed us to record a total of 47 species, belonging to the families Aphalaridae (5 species), Liviidae (5), Psyllidae (24) and Triozidae (13). For one additional species [*T.rhamni* (Schrank)], belonging to the family Triozidae, only records in literature are available relating to the presence of the galls it causes on the host plant; therefore, its presence in Luxembourg is highly probable, but needs to be confirmed by observations of psyllid specimens. The total dataset originating from the analysed 1999/2000 and 2019/2020 material can be retrieved from  https://doi.org/10.15468/svfh53  (Rapisarda et al. 2021).

In the following, brief information is provided for all 48 species whose presence has been ascertained or, as reported above for *T.rhamni*, are very likely in Luxembourg. Depicted are head structures (Figs 1, 2, 3, 4), forewings (Figs 5, 6, 7, 8), female and male terminalia (Figs 9, 10, 11, 12, 13, 14, 15, 16).

### Aphalaridae

**Aphalarasp.gr.polygoni Foerster, 1848**

(Figs 1, 5, 9)

Findings in Luxembourg. Personal field collection by the authors: Oesling: Fléiber (1 ♀, 20.VIII.2019, by general sweeping with net, including clover, *Phacelia* sp. and *Atriplex* sp.), Fussekaul (1 ♀, 22.VIII.2019, on *Rumex* sp.), Goebelsmuehle (2 ♀♀, 20.VIII.2019, on *Rumex* sp.); West Gutland: Eschdorf (2 ♀♀, 14.VIII.2019, on *Rumex* sp.).

Material studied in the MNHNL collection: Minette: Kockelscheuer, Conter Jans Boesch (1 ♀, 2.IX - 27.IX.1999, Malaise).

New record for Luxembourg: no member of the genus *Aphalara* Foerster has been recorded in Luxembourg so far; the identification of this taxon needs to be validated by the investigation of male specimens.

According to Burckhardt and Lauterer (1997b) and Cho et al. (2017b), species of this group have a Palaearctic distribution and live on plants of the family Polygonaceae, overwintering on shelter plants. Due to a high similarity of species within the genus *Aphalara* Foerster and the recent clarification of their taxonomic status (Burckhardt and Lauterer 1997b), many records of *A.polygoni* in various countries are doubtful. Hence, we decided to be more conservative by referring to the species group *A.polygoni* instead.

***Craspedoleptanebulosa* (Zetterstedt, 1828)**

(Figs 1, 5, 9, 13)

Findings in Luxembourg. Personal field collection by the authors: West Gutland: Arsdorf (7 ♂♂, 5 ♀♀, 8.VI.2020, on *Epilobiumangustifolium*).

New record for Luxembourg.

Geographical distribution. Apart from North Africa, *C.nebulosa* is widely distributed in the Holarctic Region, from Far East Russia to nearly all north and central Europe, through Central Asia and was recorded also in North America (Canada, USA) (Hodkinson and White 1979, Burckhardt 1983, Hodkinson 1988, Ossiannilsson 1992, Conci et al. 1993, Conci et al. 1996, Tishetshkin 2007, O’Connor and Malumphy 2011, Percy et al. 2012, Drohojowska and Burckhardt 2014, Ouvrard et al. 2015, den Bieman et al. 2019, Drohojowska and Klasa 2019).

Biology. Monophagous on *Epilobiumangustifolium* L. (Onagraceae) (Hodkinson and White 1979, Burckhardt 1983, Hodkinson 1988, Ossiannilsson 1992, Conci et al. 1993, Conci et al. 1996, Tishetshkin 2007, Percy et al. 2012), on which it performs one generation per year and overwinters as a nymph (usually 4^th^ instar) on the roots. This psyllid causes both foliar (optional) and root galls. The former consist in downward foldings of the leaf margin, which in June-July, protect the eggs (arranged in a long line along the lower margin) and subsequently the young nymphs; these very soon (already at the 1^st^ or 2^nd^ instar) migrate to the roots, where they overwinter (Sampò 1975). Root galls are large, thread-like and tangled (Lauterer 1976).

***Craspedoleptanervosa* (Foerster, 1848)**

(Figs 1, 5, 9)

Findings in Luxembourg. Material studied in the MNHNL collection: East Gutland: Godbrange, Schléidelbierg (1 ♀, 27.V - 9.VI.1999, Malaise).

New record for Luxembourg.

Geographical distribution. Eurosiberian distribution, from Yakutskiya and Siberia, through Mongolia, Central European Russia and Iraq, to nearly all Europe (Wagner and Franz 1961, Loginova 1968, Loginova 1974, Hodkinson and White 1979, Burckhardt 1983, Rapisarda 1985, Burckhardt and Kofler 1991, Ossiannilsson 1992, Conci et al. 1993, Conci et al. 1996, Lauterer 1994, Lauterer 2001, Seljak 2006, Ripka 2009, Malenovský et al. 2011, O’Connor and Malumphy 2011, Malenovský and Lauterer 2012, Kanturski and Drohojowska 2013, Ouvrard et al. 2015, Serbina et al. 2015, den Bieman et al. 2019, Drohojowska and Klasa 2019).

Biology. Oligophagous on plants of the genera *Achillea* L. (especially *A.millefolium* L., but also *A.gerberi* Willd., *A.micrantha* Willd., *A.nobilis* L., *A.ptarmica* L.) and *Cirsium* Miller [especially *C.arvense* (L.) Scop.] (Asteraceae) (Wagner and Franz 1961, Loginova 1968, Loginova 1974, Hodkinson and White 1979, Burckhardt 1983, Burckhardt and Kofler 1991, Ossiannilsson 1992, Conci et al. 1993, Lauterer 1994, Conci et al. 1996, Lauterer 2001, Seljak 2006, Ripka 2008, Hodkinson 2009, Malenovský et al. 2011, Malenovský and Lauterer 2012). It develops one generation a year and overwinters as a nymph on the roots of its host plants (Lauterer 1991).

***Craspedoleptasubpunctata* (Foerster, 1848)**

(Figs 1, 5, 9, 13)

Findings in Luxembourg. Personal field collection by the authors: West Gutland: Arsdorf (4 ♂♂, 3 ♀♀, 3 nymphs, 8.VI.2020, on *Epilobiumangustifolium*).

New record for Luxembourg.

Geographical distribution. Similar to *Craspedoleptanebulosa*, it is widely distributed in the north and central part of the Holarctic Region: from Far East Russia, through Central Asia, to nearly all north and central Europe and North America (Canada, USA) (Hodkinson and White 1979, Hodkinson 1988, Ossiannilsson 1992, Conci et al. 1993, Burckhardt 1994b, Conci et al. 1996, Tishetshkin 2007, Gertsson 2010, O’Connor and Malumphy 2011, Serbina et al. 2015, Drohojowska and Klasa 2019, den Bieman et al. 2019).

Biology. Monophagous on *Epilobiumangustifolium* L. (Onagraceae). According to Lauterer and Baudys (1968), adults fly in late May/early June and lay eggs on leaves and stems in June; the 1^st^ instar nymphs migrate to the roots, where they cause small galls, which grow with the development of the subsequent nymphal stages (2^nd^-4^th^ instars); the 4^th^ instar nymphs appear in late August – early September and spend the winter in the root galls; during the following May, these nymphs abandon the galls, climb up to the aerial part of the plant and produce the 5^th^ instar nymphs, which in turn give rise to the new adults.

The gall caused by *C.subpunctata* is a conglomerate of tangled, enlarged and deformed rootlets, which reaches its maximum size (up to about one centimetre) in mid-September, when it contains one to three 4^th^ instar nymphs.

***Rhinocolaaceris* (Linnaeus, 1758)**

(Figs 1, 5, 9, 13)

Findings in Luxembourg. Personal field collection by the authors: Oesling: Marnach (1 ♀, 2.VI.2020, on *Pyruscommunis*, occasional plant); West Gutland: Dondelange (1 ♂, 3 ♀♀, 28.V.2020, on *Acercampestre*), Esch-sur-Sûre (13 ♂♂, 24 ♀♀, 15.V.2020, on *A.campestre*; 3 ♂♂, 4 ♀♀, 15.V.2020, on *Acer* sp.), Préitzerdaul (1 ♂, 1 ♀, 20.V.2020, on *Populustremula*, occasional plant); Moselle: Erpeldange (1 ♀, 7.V.2020, on *Crataegusmonogyna*, occasional plant), Mondorf-les-Bains (8 ♂♂, 12 ♀♀, 18.V.2020, on *A.campestre*; 1 ♀, 18.V.2020, on *Salix* sp., occasional plant), Moutfort (3 ♂♂, 3 ♀♀, 1.VI.2020, on *Acerplatanoides*), Remich (9 ♂♂, 18 ♀♀, 8.V.2020, on *Acer* sp.).

Material studied in the MNHNL collection: East Gutland: Niederanven, Aarnescht (1 ♀, 9.VI - 25.VI.1999, Malaise).

New record for Luxembourg.

Geographical distribution. Widely distributed in the central-western part of the Palaearctic Region, with records from middle Asia, Caucasian Region (Armenia, Georgia), Turkey, nearly all Europe (except Ireland and Iberian Peninsula) and North Africa (Tunisia) (Hodkinson and White 1979, Burckhardt 1988b, Burckhardt and Lauterer 1989, Ossiannilsson 1992, Burckhardt and Önuçar 1993, Conci et al. 1993, Conci et al. 1996, Seljak 2006, Ripka 2008, Gertsson 2010, Lauterer 2011, Malenovský et al. 2011, Kanturski and Drohojowska 2013, Ouvrard et al. 2015, Serbina et al. 2015, Drohojowska and Klasa 2019).

Biology. Strictly oligophagous on *Acer* spp. (Sapindaceae). Host plants: *Acercampestre* L., *A.platanoides* L., *A.pseudoplatanus* L., *A.tataricus* L. (Hodkinson and White 1979, Burckhardt 1983, Burckhardt 1984, Burckhardt 1988a, Burckhardt and Lauterer 1989, Ossiannilsson 1992, Conci et al. 1993, Conci et al. 1996, Lauterer 2001, Burckhardt 2002, Seljak 2006, Ripka 2008, Hodkinson 2009, Malenovský et al. 2011). Adults were also collected on *Betulapendula* Roth (Betulaceae), *Buxussempervirens* L. (Buxaceae) and *Ulmus* spp. (Ulmaceae) (Burckhardt 1988a, Ripka 2008, Kanturski and Drohojowska 2013) which are “occasional plants”. *Rhinocolaaceris* performs one generation per year and, according to Lauterer (1991), overwinters in the egg stage and spends the summer in parapausa on its host plants.

### Liviidae

***Camarotoscenaspeciosa* (Flor, 1861)**

Findings in Luxembourg. This species has not been found by the authors in Luxembourg so far. Nevertheless, findings of its leaf deformations produced on *Populus* sp. and *P.nigra* were reported by Lambinon et al. (2012) (Eastern Gutland: Ettelbruck) and the occurrence of its nymphs in the above galls was described by Burton et al. (2019) (Eastern Gutland: Mersch).

Geographical distribution. Present in almost all of Europe, *C.speciosa* extends its distribution also to the Middle East (Iraq, Turkey) and Central Asia (Caucasian Region, Mongolia, former south European Russia, Tadzhikistan, Turkmenistan, Xinjiang Chinese autonomous region) (Baeva and Kankina 1971, Hodkinson and White 1979, Ossiannilsson 1992, Burckhardt and Önuçar 1993, Conci et al. 1993, Rapisarda 1994, Nokkala 1995, Conci et al. 1996, Burckhardt and Mifsud 2003, Drees 2005, Seljak 2006, Ripka 2008, Mustafa et al. 2014, Serbina et al. 2015, den Bieman et al. 2019).

Biology. Oligophagous on plants of the genus *Populus* L. (amongst which, *P.alba* L., *P.nigra* L. and *P.tremula* L. are important hosts in Europe) (Salicaceae) (Hodkinson and White 1979, Ossiannilsson 1992, Burckhardt and Mifsud 2003, Tomasi 2003, Seljak 2006, Ripka and Kiss 2008, Mustafa et al. 2014), *C.speciosa* performs only one annual generation and overwinters as adults on shelter plants (conifers). Leaf galls produced by this psyllid, consisting in a winding up of the leaf margin (sometimes turning to reddish), are reported in older literature (Houard 1908, Houard 1909, Houard 1913, Ross and Hedicke 1927, Buhr 1964, Buhr 1965); however, *C.speciosa* does not seem to produce significant damage to infested poplar plants.

***Liviajunci* (Schrank, 1789)**

(Figs 1, 5, 9, 13)

Findings in Luxembourg. Material studied in the MNHNL collection: Oesling: Eselborn, Bréichen (2 ♂♂, 2 ♀♀, 24.VIII - 22.IX.2000; 1 ♂, 19.X - 7.XI.2000, Malaise), Hoffelt, Sporbech (4 ♂♂, 7 ♀♀, 22.IX - 19.X.2000, Malaise), Sonlez, Pamer (10 ♂♂, 6 ♀♀, 3.VIII - 24.VIII.2000; 10 ♂♂, 3 ♀♀, 24.VIII - 22.IX.2000; 10 ♂♂, 6 ♀♀, 22.IX - 19.X.2000, Malaise); East Gutland: Wilferdange, Conzefenn (1 ♂, 3 ♀♀, 3.VIII - 24.VIII.2000; 1 ♂, 3 ♀♀, 22.IX - 19.X.2000, Malaise).

No explicit mention exists in literature on previous findings of this species in Luxembourg, though deformations it causes to inflorescences of *Juncusarticulatus* L. are reported by Lambinon and Schneider (2004) and by Schneider (2016) (West Gutland: Hollenfels; Moselle: Remerschen). These authors, however, do not report any findings of this psyllid in or nearby the galls. Therefore, the present records represent the first direct observations of *L.junci* in Luxembourg.

Geographical distribution. *Liviajunci* is spread all over the Palaearctic Region and has been found in nearly all of Europe, north Africa (Algeria, Morocco), the Middle East (Cyprus, Iran, Lebanon, Turkey) and central, south and eastern Asia (Caucasian Region, India, Kazakhstan, Kyrgyzstan, Primorsky Krai, Siberia, Tadzhikistan, Turkmenistan) (Hodkinson and White 1979, Ossiannilsson 1992, Burckhardt and Lauterer 1993, Burckhardt and Önuçar 1993, Conci et al. 1993, Conci et al. 1996, Zeidan-Gèze and Burckhardt 1998, Hodkinson and Bird 2000, Seljak 2006, Ripka 2008, Malenovský et al. 2011, O’Connor and Malumphy 2011, Burckhardt et al. 2018, den Bieman et al. 2019).

Biology. Oligophagous on many species of the genus *Juncus* L. (Juncaceae) (Hodkinson and White 1979, Burckhardt 1983, Burckhardt 1988a, Hodkinson 1986, Ossiannilsson 1992, Conci et al. 1993, Conci et al. 1996, Burckhardt 2005, Zeidan-Gèze and Burckhardt 1998, Hodkinson and Bird 2000, Seljak 2006, Ripka 2008, Drohojowska 2009a, Hodkinson 2009, Drohojowska 2009b, Malenovský et al. 2011, Burckhardt et al. 2018). It performs one generation per year and overwinters as adults on shelter plants (conifers). During spring, adults fly back to their host plants, on which they lay eggs. After hatching, nymphs move to the young shoots and start producing characteristic galls by transforming the inflorescences into masses of reddish small leaflets, very close to each other due to the sharp shortening of internodes (Darboux and Houard 1901, Kieffer 1901, Houard 1908, Houard 1909, Houard 1913, Ross and Hedicke 1927, Buhr 1964, Buhr 1965).

***Psyllopsisfraxini* (Linnaeus, 1758)**

(Figs 1, 5, 9, 13)

Findings in Luxembourg. Galls produced by this psyllid on *Fraxinusexcelsior* L. are reported by Lambinon and Schneider (2004) (Oesling: Allerborn, Beiler, Biwisch, Clervaux, Hautbellain, Hosingen, Kiirchermillen, between Moersdorf and Langsur [*sic*!], Vianden; Moselle: Ahn, Bous, Deisermillen, Fausermillen, Grevenmacher, Mertert, Mondorf-les-Bains, Stadtbredimus, Wasserbillig; Minette: Bettembourg, Belvaux, Dudelange, Esch-sur-Alzette, Gantenbeinsmühle, Hesperange, Rumelange, Schifflange, Noertzange, Oberkorn, Pétange, Prënzebierg; West Gutland: Beckerich, Bereldange, Bonnevoie, Bridel, Capellen, Cents, Grass, Haardt, Hamm, Hollerich, Howald, Kleinbettingen, Luxembourg Ville, Pulvermühle, Schleifmühle, Stadtgrund, Steinfort, Steinsel, Walferdange, Windhof; East Gutland: Beringen, Bettendorf, Betzdorf, Biirgerkräiz, Colmar-Berg, Diekirch, Ettelbruck, Gilsdorf, Hagelsdorf, Lintgen, Lorentzweiler, Marxmillen, Manternach, Moesdorf, Neudorf, Niederanven, Oetrange, Roodt-sur-Syre, Rosport, Uebersyren, Wecker, Warken). According to the same authors, the lab rearing from galls collected in some of the above locations always produced adults of *P.fraxini* .

Personal field collection by the authors: Minette: Kayl/Tetange (1 ♂, 2 ♀♀, 23.VII.2019, on *F.excelsior*).

Geographical distribution. Central Asian - European chorotype, diffused from the Himalayan Region (Uttaranchal) westwards to Iran and great parts of Europe (Hodkinson and White 1979, Ossiannilsson 1992, Burckhardt and Lauterer 1993, Conci et al. 1993, Seljak 2006, Ripka 2008, Gertsson 2010, Malenovský et al. 2011, O’Connor and Malumphy 2011, Kanturski and Drohojowska 2013, Ouvrard et al. 2015, Serbina et al. 2015, Burckhardt et al. 2018, Drohojowska and Klasa 2019, den Bieman et al. 2019). Introduced to North America (USA; Hodkinson 1988) and the Australian Region (New Zealand and Tasmania; Hollis 2004, Martoni et al. 2018).

Biology. Strictly oligophagous on ashes (*Fraxinus* L., Oleaceae). According to the literature, this psyllid is common especially on *Fraxinusexcelsior* L., but can be collected also on *F.americana* L., *F.angustifolia* Vahl, *F.aurea* Willd., *F.mandschurica* Rupr., *F.ornus* L., *F.oxycarpa* Willd., *F.pendula* (Aiton) Hoffmanns, *F.pennsylvanica* Marshall (Hodkinson and White 1979, Burckhardt 1983, Hodkinson 1988, Seljak 2006, Ripka 2008, Burrows 2012). On its host plants, *P.fraxini* performs 1-2 generations per year and overwinters in the egg stage.

This psyllid produces showy galls, widely described in literature (e.g. Darboux and Houard 1901, Houard 1908, Houard 1909, Houard 1913, Ross and Hedicke 1927, Buhr 1964, Buhr 1965, Sampò 1975), consisting in the downrolling of the leaf margin; the rolled up part becomes dilated, thickened, turgescent, conspicuously cross-linked in red and violet.

***Psyllopsisfraxinicola* (Foerster, 1848)**

(Figs 1, 5, 9, 13)

Findings in Luxembourg. Personal field collection by the authors: West Gutland: Eschdorf (1 ♀♀, 14.VIII.2019, on *Fraxinusexcelsior*; 1 ♀♀, 14.VIII.2019, on *Prunus* sp., occasional plant); Minette: Kayl/Tetange (3 ♂♂, 3 ♀♀, 23.VII.2019, on *F.excelsior*); Mosel: Bech/Kleinmacher (1 ♀, 30.VII.2019, on *F.excelsior*), Mondorf-les-Bains (1 ♂, 1 ♀, 1.VIII.2019, on *Populuscinerea*, occasional plant), Remich (1 ♂, 1.VIII.2019, on *F.excelsior*).

New record for Luxembourg.

Geographical distribution. Turanic-European-Mediterranean chorotype, diffused from Central Asia (Kazakhstan) westwards to the Caucasian Region (Armenia, Georgia), Turkey, nearly all of Europe and North Africa (Hodkinson and White 1979, Burckhardt 1983, Burckhardt 1988a, Burckhardt 1989, Burckhardt and Halperin 1992, Ossiannilsson 1992, Conci et al. 1993, Burckhardt and Önuçar 1993, Burckhardt 2005, Seljak 2006, Ripka 2008, Malumphy et al. 2009, Gertsson 2010, Malenovský et al. 2011, O’Connor and Malumphy 2011, Kanturski and Drohojowska 2013, Ouvrard et al. 2015, Serbina et al. 2015, Holzinger et al. 2017, Spodek et al. 2017, den Bieman et al. 2019). Introduced to the Americas (Canada, USA, South America; Hodkinson 1988, Percy et al. 2012, Castillo Carrillo et al. 2016) and the Australian Region (Australia, New Zealand; Hollis 2004, Percy et al. 2012, Martoni et al. 2018).

Biology. Strictly oligophagous on ashes (*Fraxinus* L., Oleaceae). Especially common on *Fraxinusexcelsior* L. According to literature, this psyllid has been collected also on *F.angustifolia* Vahl, *F.dipetala* Hook. & Am., *F.ornus* L., *F.syriaca* Boiss. (Hodkinson and White 1979, Burckhardt 1983, Burckhardt 1988a, Burckhardt 1989, Hodkinson 1988, Burckhardt 2005, Seljak 2006, Ripka 2008, Drohojowska 2009a, Percy et al. 2012, Spodek et al. 2017), but it is not sure if they are host or casual plants. On its host plants, *P.fraxinicola* performs 1-2 generations per year and overwinters in the egg stage.

***Strophingiaericae* (Curtis, 1835)**

(Figs 1, 5, 9, 13)

Findings in Luxembourg. Personal field collection by the authors: West Gutland: Brouch (4 ♀♀, 15.V.2020, on *Callunavulgaris*), Esch-sur-Sûre (1 ♂, 15.V.2020, on *C.vulgaris*). Material studied in the MNHNL collection: Oesling: Lellingen, Op Baerel (1 ♀, 8.VI - 20.VI.2000, Moericke).

New record for Luxembourg.

Geographical distribution. *Strophingiaericae* is a typical European chorotype and is widespread in almost all Europe, though less common in the Mediterranean part of the continent (Iberian Peninsula, central and south Italy, Balkan Peninsula) (Hodkinson and White 1979, Burckhardt 1983, Ossiannilsson 1992, Conci et al. 1993, Conci et al. 1996, Burckhardt 2005, Seljak 2006, Gertsson 2010, O’Connor and Malumphy 2011, Serbina et al. 2015, den Bieman et al. 2019).

Biology. Oligophagous on Ericaceae, with *Callunavulgaris* (L.) Hull as main host plant (Hodkinson and White 1979, Burckhardt 1983, Ossiannilsson 1992, Conci et al. 1993, Conci et al. 1996, Seljak 2006), but also recorded on *Ericacinerea* L. and *Vacciniumuliginosum* L. (Hodkinson and White 1979, Hodkinson 1981, Burckhardt 2005). Eventual other plants should be considered as occasional hosts. Winter is spent by this psyllid as a nymph on the host plants. In north England, where the biology of *S.ericae* has been thoroughly investigated (Hodkinson 1973a, Hodkinson 1973b, Parkinson and Whittaker 1975), this psyllid develops its cycle in one year at low altitude and in two years at higher altitudes, thus presenting two physiological "races", characterised also by very small morphological differences. Studies on population dynamics of *S.ericae* are reported by Hodkinson (1973b).

### Psyllidae

Figs 2, 6, 10

***Arytainagenistae* (Latreille, 1804)**

(Figs 2, 6, 10, 14)

Findings in Luxembourg. Personal field collection by the authors: Oesling: Arsdorf (22 ♂♂, 39 ♀♀, 22.VIII.2019, on *Cytisusscoparius*), Berlé (1 ♂, 2 ♀♀, 20.VIII.2019, on *C.scoparius*); West Gutland: Bertrange (1 ♀, 7.VI.2020, on *C.scoparius*), Bridel (7 ♂♂, 11 ♀♀, 6.VIII.2019, on *C.scoparius*), Brouch (1 ♂, 1 ♀, 6.IX.2019, by general sweeping with net); Minette: Kayl/Tetange (1 ♀, 23.VII.2019, on *C.scoparius*).

Material studied in the MNHNL collection: Oesling: Basbellain, Klengelbaach (1 ♀, 24.VIII - 22.IX.2000; 1 ♀, 22.IX - 19.X.2000, Malaise), Eselborn, Bréichen (1 ♀, 3.VIII - 24.VIII.2000, Malaise), Goebelsmühle (3 ♂♂, 2 ♀♀, 13.IV - 27.IV.2000; 2 ♂♂, 5 ♀♀, 25.V - 8.VI.2000; 8 ♂♂, 4 ♀♀, 20.VI - 6.VII.2000; 13 ♂♂, 13 ♀♀, 6.VII - 3.VIII.2000; 1 ♂, 2 ♀♀, 3.VIII - 24.VIII.2000; 4 ♂♂, 1 ♀, 24.VIII - 22.IX.2000; 1 ♂, 2 ♀♀, 22.IX - 19.X.2000, Malaise), Hoscheid, Molberlay (2 ♂♂, 1 ♀, 13.IV - 27.IV.2000; 4 ♂♂, 11 ♀♀, 20.VI - 6.VII.2000; 11 ♂♂, 14 ♀♀, 6.VII - 3.VIII.2000; 4 ♂♂, 8 ♀♀, 3.VIII - 24.VIII.2000; 2 ♂♂, 5 ♀♀, 24.VIII - 22.IX.2000; 1 ♀, 22.IX - 19.X.2000, Malaise), Lellingen, Op Baerel (2 ♂♂, 8.VI - 20.VI.2000; 3 ♂♂, 20.VI - 6.VII.2000, Malaise).

New record for Luxembourg.

Geographical distribution. Widespread in Europe and adventive in the USA, Canada (Nova Scotia) and New Zealand (Hodkinson and White 1979, Ossiannilsson 1992, Hodkinson and Hollis 1987, Hodkinson 1988, Conci et al. 1993, Conci et al. 1996, Seljak 2006, Percy et al. 2012, Serbina et al. 2015, Bleach 2019, den Bieman et al. 2019). Some of the geographical reports need confirmation, due to likely confusion with related species.

Biology. Probably oligophagous on plants of the genus *Cytisus* L. [especially *C.scoparius* (L.) Link] (Fabaceae) (Hodkinson and White 1979, Burckhardt 1983, Hodkinson and Hollis 1987, Hodkinson 1988, Ossiannilsson 1992, Conci et al. 1993, Conci et al. 1996, Burckhardt 2005, Seljak 2006, Syrett et al. 2007, Percy et al. 2012); its reports on other Genistinae (e.g. *Chamaecytisus* spp., *Genistatinctoria* L. and *Ulexeuropaeus* L.) are doubtful or need to be confirmed. This psyllid performs 2-3 (in southern Europe maybe more) generations per year, with an almost continuous development; it spends the winter in all developmental stages (especially as an adult) on its host plants.

***Arytainillaspartiophila* (Foerster, 1848)**

(Figs 2, 6, 10, 14)

Findings in Luxembourg. Personal field collection by the authors: Oesling: Urspelt (3 ♂♂, 16 ♀♀, 2.VI.2020, on *Cytisusscoparius*); West Gutland: Arsdorf (30 ♂♂, 20 ♀♀, 20.V.2020, on *C.scoparius*; 4 ♀♀, 20.V.2020, by general sweeping), Bertrange (1 ♂, 24 ♀♀, 7.VI.2020, on *C.scoparius*), Brouch (40 ♂♂, 46 ♀♀, 15.V.2020, on *C.scoparius*), Dondelange (1 ♂, 1 ♀, 28.V.2020, by general sweeping), Elvange/Schweich (1 ♀, 28.V.2020, on *Prunusspinosa*, occasional plant; 1 ♂, 28.V.2020, on *Salixviminalis*, occasional plant), Esch-sur-Sûre (11 ♂♂, 12 ♀♀, 15.V.2020, on *C.scoparius*; 4 ♂♂, 7 ♀♀, 15.V.2020, on *P.spinosa*, occasional plant), Goesdorf/Bockholtz (1 ♂, 1 ♀, 20.V.2020, on *Acerpseudoplatanus*, occasional plant; 2 ♂♂, 6 ♀♀, 20.V.2020, on *Sonchus* sp., occasional plant), Hobscheid (4 ♂♂, 6 ♀♀, 28.V.2020, on *C.scoparius*; 1 ♂, 1 ♀, 28.V.2020, on *Malus* sp., occasional plant), Noerdange (1 ♀, 28.V.2020, on *Pyruscommunis*, occasional plant), Strassen (19 ♂♂, 4 ♀♀, 28.V.2020, on *C.scoparius*; 3 ♂♂, 8.VI.2020, on *C.scoparius*); Minette: Belvaux (8 ♂♂, 3 ♀♀, 7.V.2020, on *C.scoparius*), Kayl (1 ♀, 20.V.2020, on *Malus* sp., occasional plant); Mosel: Mondorf (1 ♂, 18.V.2020, on *Acercampestre*, occasional plant; 1 ♂, 18.V.2020, on *Crataegusmonogyna*, occasional plant).

Material studied in the MNHNL collection: Oesling: Goebelsmühle (313 ♂♂, 594 ♀♀, 11.V - 25.V.2000; 384 ♂♂, 611 ♀♀, I nymph, 25.V - 8.VI.2000; 5 ♂♂, 6 ♀♀, 20.VI - 6.VII.2000; 4 ♂♂, 5 ♀♀, 6.VII - 3.VIII.2000; 1 ♂, 1 ♀, 24.VIII - 22.IX.2000; 1 ♂, 22.IX - 19.X.2000, Malaise), Hoscheid, Molberlay (1 ♀, 20.VI - 6.VII.2000, Malaise), Lellingen, Op Baerel (88 ♂♂, 87 ♀♀, 25.V - 8.VI.2000; 25 ♂♂, 33 ♀♀, 8.VI - 20.VI.2000; 2 ♂♂, 20.VI - 6.VII.2000, Malaise); West Gutland: Capellen, Werwelslach (1 ♂, 2 ♀♀, 27.V - 9.VI.1999, Malaise); East Gutland: Godbrange, Schléidelbierg (3 ♂♂, 7 ♀♀, 27.V - 9.VI.1999, Moericke), Niederanven, Aarnescht (3 ♂♂, 2 ♀♀, 27.V - 9.VI.1999, Malaise); Minette: Schifflange, Kayl, Brucherbierg (3 ♂♂, 5 ♀♀, 27.V - 9.VI.2000, Malaise); Moselle: Canach, Wéngertsbierg (6 ♀♀, 27.V - 9.VI.1999; 1 ♀, 9.VI - 25.VI.1999, Malaise).

New record for Luxembourg.

Geographical distribution. Widespread in central-western and southern Europe (Hodkinson and White 1979, Ossiannilsson 1992, Conci et al. 1993, Conci et al. 1996, Malumphy et al. 2009, O’Connor and Malumphy 2011, den Bieman et al. 2019). Introduced to North America (Canada, USA), Australia and New Zealand (Hodkinson 1988, Percy et al. 2012).

Biology. *Arytainillaspartiophila* is monophagous on *Cytisusscoparius* (L.) Link (Fabaceae), on which it spends its entire life cycle, performing a single generation per year and overwintering as egg. In the Northern Hemisphere, adults start to fly in mid-April and occur on the plants till the first half of June; from the second half of May, males start to decline in number and populations of this psyllid become female-biased (Wheeler 2017). For its exclusive monophagy on *C.scoparius* and the large populations, this psyllid can build up on Scotch broom in many European countries (especially in Great Britain) causing substantial damage; it has been artificially introduced and released for biological control in exotic habitats (such as California and New Zealand) where its host plant became invasive (Syrett et al. 2007, Hogg et al. 2015).

***Cacopsyllaaffinis* (Löw, 1880)**

(Figs 2, 6, 14)

Findings in Luxembourg. Material studied in the MNHNL collection: Oesling: Lellingen, Op Baerel (1 ♂, 25.V - 8.VI.2000, Malaise).

In the absence of male specimens, it is impossible to morphologically distinguish this species from *Cacopsyllamelanoneura* (Foerster); for this reason, collections in Luxembourg of female specimens, here attributed to the more common species *C.melanoneura*, could also refer to *C.affinis*.

New record for Luxembourg.

Geographical distribution. Distributed in most of Europe, eastwards to Turkey and the Caucasian Region (Hodkinson and White 1979, Burckhardt 1983, Burckhardt 1988a, Ossiannilsson 1992, Conci et al. 1993, Burckhardt and Önuçar 1993, Conci et al. 1996, Seljak 2006, Tedeschi et al. 2008, Ripka 2008, Ripka 2009, Malenovský et al. 2011, den Bieman et al. 2019).

Biology. Oligophagous on hawthorns (*Crataegus* spp.) (Rosaceae) (Burckhardt 1983, Burckhardt 1988a, Ossiannilsson 1992, Conci et al. 1993, Conci et al. 1996, Seljak 2006, Hodkinson 2009, Malenovský et al. 2011), *C.affinis* performs one generation per year and overwinters as adult on shelter plants, especially conifers and Fagaceae.

Economic significance. A controversial phytosanitary importance is attributed to this species as potential vector of phytopathogenic microorganisms, especially ‘*Candidatus* Liberibacter europaeus’ (Tedeschi et al. 2009, Camerota et al. 2012) which should be considered, however, as an endophyte rather than a real pathogen (Raddadi et al. 2011).

***Cacopsyllaambigua* (Foerster, 1848)**

(Figs 2, 6, 10, 14)

Findings in Luxembourg. Personal field collection by the authors: West Gutland: Brouch (3 ♂♂, 8 ♀♀, 15.V.2020, on *Salixcaprea*; 1 ♀, 15.V.2020, on *Salixviminalis*), Elvange/Schweich (2 ♂♂, 28.V.2020, on *S.viminalis*), Strassen (3 ♂♂, 5 ♀♀, 27.IV.2020, on *S.caprea*); East Gutland: Rodenburg (1 ♀, 27.V.2020, on *S.caprea*).

Material studied in the MNHNL collection: East Gutland: Wilferdange, Conzefenn (1 ♀, 6.VII - 3.VIII.2000, Malaise).

New record for Luxembourg.

Geographical distribution. *Cacopsyllaambigua* is an Eurasian chorotype, especially having a wide distribution in Europe (Hodkinson and White 1979, Burckhardt 1988a, Ossiannilsson 1992, Conci et al. 1993, Conci et al. 1996, Lauterer and Burckhardt 1997, Labina 2006, Seljak 2006, Labina 2008, Ripka 2008, Inoue 2010, O’Connor and Malumphy 2011, Kanturski and Drohojowska 2013, Ouvrard et al. 2015, Serbina et al. 2015, Zendedel et al. 2016, Drohojowska and Klasa 2019, den Bieman et al. 2019).

Biology. Strictly oligophagous on *Salix* spp. (Salicaceae), being reported in literature from *S.alba* L., *S.atrocinerea* Brot., *S.aurita* L., *S.caprea* L., *S.cinerea* L., *S.elaeagnos* Scop., *S.incana* Schrank, *S.lapponum* L., *S.purpurea* L. and *S.viminalis* L. (Hodkinson and White 1979, Ossiannilsson 1992, Conci et al. 1993, Conci et al. 1996, Lauterer and Burckhardt 1997). On its host plants, it performs the entire developmental cycle, showing one or two generations per year and overwintering as egg or, according to Lauterer (1976), as 1^st^ or 2^nd^ instar nymph.

***Cacopsyllacrataegi* (Schrank, 1801)**

(Figs 2, 6, 10, 14)

Findings in Luxembourg. Personal field collection by the authors: West Gutland: Strassen (1 ♂, 2 ♀♀, 27.IV.2020, on *Crataegusmonogyna*).

Material studied in the MNHNL collection: West Gutland: Capellen, Werwelslach (1 ♀, 18.V - 27.V.1999, Moericke); East Gutland: Godbrange, Schléidelbierg (4 ♂♂, 1 ♀, 6.IV - 22.IV.1999, Malaise); Minette: Niedercorn, Giele Botter (2 ♂♂, 25.VI - 12.VII.1999, Malaise); Moselle: Canach, Wéngertsbierg (1 ♂, 1 ♀, 22.IV - 11.V.1999; 1 ♂, 11.V - 27.V.1999; 1 ♂, 27.V - 9.VI.1999, Malaise).

New record for Luxembourg.

Geographical distribution. Widely distributed in the Palaearctic Region, from central-south Asia (west Himalayan Region, India, Iran, Caucasian Region) to nearly all parts of Europe and North Africa (Algeria, Morocco) (Hodkinson and White 1979, Burckhardt 1988a, Burckhardt 1989, Ossiannilsson 1992, Conci et al. 1993, Burckhardt and Lauterer 1993, Conci et al. 1996, Seljak 2006, Ripka 2008, Ripka and Kiss 2008, Gertsson 2010, Malenovský et al. 2011, O’Connor and Malumphy 2011, Kanturski and Drohojowska 2013, Oettl and Schlink 2015, Ouvrard et al. 2015, Serbina et al. 2015, Burckhardt et al. 2018, Drohojowska and Klasa 2019, den Bieman et al. 2019).

Biology. Strictly oligophagous on hawthorns (*Crataegus* spp.) (Rosaceae), *C.crataegi* is mainly reported from *Crataegusmonogyna* Jacq. and *C.oxyacantha* L. (Ossiannilsson 1992, Conci et al. 1993, Conci et al. 1996), being found also on other species, especially in Asia, such as *C.coccinea* L. or *C.pentagyna* Waldst. & Kit. ex Willd. (Ossiannilsson 1992). Besides its host plants and various occasional species, this psyllid can be found frequently on other Rosaceae, such as *Malus* spp., *Mespilus* spp. or *Sorbus* spp. (Burckhardt 1989, Ripka 2008, Oettl and Schlink 2015). *Cacopsyllacrataegi* performs only one generation per year and overwinters as an adult on shelter plants (conifers).

***Cacopsyllamali* (Schmidberger, 1836)**

(Figs 2, 6, 10, 14)

Findings in Luxembourg. Personal field collection by the authors: West Gutland: Dondelange (4 ♂♂, 2 ♀♀, 28.V.2020, on *Malus* sp.); Minette: Kayl (3 ♂♂, 6 ♀♀, 20.V.2020, on *Malus* sp.).

No explicit mention exists in literature on findings of this insect in Luxembourg, though the leaf deformations it produces on *Malus* sp. are reported by Lambinon and Schneider (2004) (West Gutland: Kirchberg). The authors, however, do not report any finding of this psyllid in or nearby the galls. Therefore, the present record represents the first direct observation of *C.mali* in Luxembourg.

Geographical distribution. *Cacopsyllamali* is widely distributed in the Eurasian Region: though not properly recorded in central Asia, it seems to occur from the Russian Far East, Japan and the Korean Peninsula to most of Europe (Hodkinson and White 1979, Burckhardt 1983, Kwon 1983, Burckhardt 1988b, Hodkinson 1988, Ossiannilsson 1992, Conci et al. 1993, Burckhardt and Önuçar 1993, Conci et al. 1996, Seljak 2006, Ripka 2008, Malumphy et al. 2009, Inoue 2010, Lauterer 2011, Malenovský et al. 2011, O’Connor and Malumphy 2011, Kanturski and Drohojowska 2013, Serbina et al. 2015, Drohojowska and Klasa 2019, den Bieman et al. 2019). It is known also from the Nearctic (USA, Canada) and Afrotropical (South Africa) Regions, as well as from Australia (Burckhardt 1988b, Hodkinson 1988, Burckhardt 1994a, Wheeler and Hoebeke 2005, Inoue 2010), where it likely has been introduced.

Biology. Strictly oligophagous on various species of the genus *Malus* Mill. (Rosaceae). In Europe, it can be found mainly on *M.domestica* Borkh. and *M.sylvestris* Mill. (Hodkinson and White 1979, Ossiannilsson 1992, Conci et al. 1993, Burckhardt 1994a, Conci et al. 1996, Ripka 2008, Inoue 2010). *Malusasiatica* Nakai, *M.baccata* (L.) Borkh. and *M.transitoria* (Batalin) C.K. Schneid. are reported as host plants of this psyllid in Asia (Kwon 1983, Li 2011). *Cacopsyllamali* performs one generation per year and overwinters as egg on its host plants. It often causes leaf alterations, corrugations and distortions (Buhr 1964, Buhr 1965). Similar to other species of the genus *Cacopsylla*, it may show a typical summer migration: part of the adults, which fed on the host plant for 2-3 weeks after emergence, migrate to other species of trees or shrubs, to re-immigrate to the host plants in September. Adults show a summer reproductive "parapause", which ends in autumn with a reactivation with their oogenesis (Lauterer 1991).

Economic significance. *Cacopsyllamali* is a secondary pest of apple trees in central Europe. In spite of old reports as a very harmful species, the damage it causes to crops is usually negligible. In recent studies, ‘*Candidatus* Phytoplasma mali’, the etiological agent of the Apple Proliferation (AP) disease, has been detected also in various psyllid species, including *C.mali*, different from the two known vectors of this pathogen [*Cacopsyllamelanoneura* (Foerster) and *C.picta* (Foerster)] (Miñarro et al. 2016); yet the real potential of *C.mali* to transmit the disease is still unclear and warrants further investigation.

***Cacopsyllamelanoneura* (Foerster, 1848)**

(Figs 2, 6, 10, 14)

Findings in Luxembourg. Personal field collection by the authors: Oesling: Marnach (9 ♂♂, 6 ♀♀, 2.VI.2020, on *Crataegusmonogyna*); West Gutland: Arsdorf (1 ♂, 7 ♀♀, 1 nymph, 20.V.2020, on *C.monogyna*), Dondelange (4 ♂♂, 2 ♀♀, 28.V.2020, on *C.monogyna*), Elvange/Schweich (2 ♂♂, 2 ♀♀, 28.V.2020, on *C.monogyna*; 4 ♀♀, 28.V.2020, on *Prunusspinosa*, occasional plant), Esch-sur-Sûre (1 ♂, 1 ♀, 15.V.2020, on *Acer* sp., occasional plant), Préitzerdaul (1 ♂, 20.V.2020, on *C.monogyna*), Strassen (1 ♂, 3 ♀♀, 27.IV.2020, on *C.monogyna*), Useldange (1 ♂, 1 ♀, 28.V.2020, on *C.monogyna*); East Gutland: Bettendorf (1 ♀, 27.V.2020, by general sweeping with net, from *Salix* sp. and *Populus* sp., occasional plants); Moselle: Elvange/Burmerange (3 ♂♂, 5 ♀♀, 19.V.2020, on *C.monogyna*; 1 ♀, 19.V.2020, by general sweeping with net), Erpeldange (6 ♂♂, 3 ♀♀, 7.V.2020, on *C.monogyna*), Mondorf (4 ♂♂, 5 ♀♀, 18.V.2020, on *C.monogyna*; 1 ♀, 28.V.2020, on *Acercampestre*, occasional plant; 1 ♀, 28.V.2020, by general sweeping with net).

Material studied in the MNHNL collection: Oesling: Basbellain, Klengelbaach (1 ♀, 22.IX - 19.X.2000, Malaise), Lellingen, Op Baerel (3 ♂♂, 8 ♀♀, 25.V - 8.VI.2000, Malaise); West Gutland: Bertrange, Brill (8 ♂♂, 14 ♀♀, 15.III - 19.III.1999, Moericke), Capellen, Werwelslach (2 ♂♂, 1 ♀, 13.IV - 22.IV.1999; 1 ♀, 22.IV - 28.IV.1999; 2 ♀♀, 18.V - 27.V.1999; 1 ♂, 2 ♀♀, 27.V - 9.VI.1999, Malaise); East Gutland: Godbrange, Schléidelbierg (44 ♂♂, 37 ♀♀, 6.IV - 22.IV.1999; 10 ♂♂, 2 ♀♀, 22.IV - 11.V.1999; 8 ♂♂, 11 ♀♀, 18.V - 27.V.1999; 23 ♂♂, 23 ♀♀, 27.V - 9.VI.1999; 2 ♂♂, 5 ♀♀, 9.VI - 25.VI.1999; 1 ♂, 25.VI - 8.VII.1999, Malaise), Koedange, Poenn (1 ♀, 11.III - 15.III.1999, Moericke), Niederanven, Aarnescht (5 ♂♂, 4 ♀♀, 22.IV - 11.V.1999; 2 ♀♀, 11.V - 27.V.1999; 1 ♀, 9.VI - 25.VI.1999, Malaise); Minette: Kockelscheuer, Conter Jans Boesch (1 ♀, 22.IV - 28.IV.1999, Malaise), Niedercorn, Giele Botter (2 ♂♂, 25.VI - 12.VII.1999, Malaise), Schifflange, Kayl, Brucherbierg (2 ♂♂, 3 ♀♀, 22.IV - 11.V.1999; 1 ♂, 1 ♀, 11.V - 27.V.1999; 2 ♂♂, 2 ♀♀, 27.V - 9.VI.1999, Malaise); Moselle: Canach, Wéngertsbierg (6 ♂♂, 5 ♀♀, 22.IV - 11.V.1999; 3 ♀♀, 11.V - 27.V.1999, Malaise).

In the absence of male specimens, it is impossible to morphologically distinguish this species from *Cacopsyllaaffinis* (Löw); for this reason, collections in Luxembourg of female specimens here attributed to *C.melanoneura* could refer to *C.affinis*. Identification tools of the two species by molecular methods have been studied by Tedeschi and Nardi (2010).

No explicit mention exists in literature on findings of *C.melanoneura* in Luxembourg, though its leaf deformations produced on *Crataegusmonogyna* Jacq. are reported by Lambinon and Schneider (2004) (Minette: Bettembourg; Moselle: Canach; West Gutland: Bonnevoie, Kleinbettingen, Steinfort; East Gutland: Oberanven), who do not report, however, any finding of this psyllid in or nearby the galls. Therefore, the present records are the first direct findings of *C.melanoneura* in Luxembourg.

Geographical distribution. Palearctic chorotype, widespread and common from the Far East Asia (Japan) to almost all Europe and North Africa (Algeria), through various confirmed reports from Central Asia [Mongolia, Russia (Irkutsk and Siberia), Tadzhikistan, Uzbekistan] (Loginova 1968, Hodkinson and White 1979, Burckhardt 1988a, Burckhardt 1989, Ossiannilsson 1992, Conci et al. 1993, Conci et al. 1996, Lauterer 1999, Baugnée et al. 2002, Seljak 2006, Ripka 2008, Malumphy et al. 2009, Gertsson 2010, Malenovský et al. 2011, O’Connor and Malumphy 2011, Chireceanu and Fătu 2012, Ouvrard et al. 2015, Drohojowska and Klasa 2019, den Bieman et al. 2019).

Biology. Oligophagous on many Rosaceae, *C.melanoneura* primarily lives on plant species of the genus *Crataegus* L. (especially frequent on *C.monogyna* Jacq. and *C.oxyacantha* L., but also reported on *C.laevigata* (Poir.) DC. and *C.maximowiczii* C.K. Schneid. (Hodkinson and White 1979, Burckhardt 1983, Burckhardt 1988a, Burckhardt 1989, Ossiannilsson 1992, Seljak 2006, Ripka 2008, Hodkinson 2009, Malenovský et al. 2011). It can live even on *Malus* spp. (*M.communis* Desf., *M.domestica* Borck.), *Mespilus* spp. (*M.germanica* L.), *Prunus* spp. (*P.armeniaca* L.) and *Pyrus* spp. (*Pyruscommunis* L.) (Ossiannilsson 1992, Conci et al. 1993, Conci et al. 1996, Seljak 2006, Ripka 2008, Hodkinson 2009, Malumphy et al. 2009). Furthermore, it can frequently be found on other occasional plants. It shows only one generation per year and overwinters in the adult stage on shelter plants (conifers); aestivation and overwintering habits of this species have been studied in detail by Pizzinat et al. (2011).

Economic significance. On hawthorn *C.melanoneura* is not a harmful species, if leaf deformations often caused by this insect are excluded, i.e. yellow to blood-red folds of the leaf margins. Nevertheless, phytosanitary importance of this species has recently been highlighted, for its ability to host and transmit phytopathogenic microorganisms of various cultivated Rosaceae (especially apple trees), such as '*Candidatus* Liberibacter europaeus' and '*Candidatus* Phytoplasma mali', the latter being the causative agent of the Apple Proliferation (AP) disease (Mayer et al. 2009, Tedeschi et al. 2009, Tedeschi et al. 2012, Camerota et al. 2012, Jarausch et al. 2012, Oettl and Schlink 2015, Kaya et al. 2016, Miñarro et al. 2016, Oppedisano et al. 2020

Given the phytosanitary importance of this species as a potential vector of phytopathogenic microorganisms, its actual distribution and pathogenicity in Luxembourg should be further investigated.

***Cacopsyllanigrita* (Zetterstedt, 1828)**

(Figs 2, 6, 10, 14)

Findings in Luxembourg. Material studied in the MNHNL collection: East Gutland: Wilferdange, Conzefenn (1 ♀, 22.IX - 19.X.2000, Malaise).

New record for Luxembourg.

Geographical distribution. Widely distributed in Europe, except its most western (e.g. Great Britain, France and Iberian Peninsula) and Mediterranean parts and reported also from the Caucasian Region and eastwards to Japan (Ossiannilsson 1992, Conci et al. 1993, Conci et al. 1996, Lauterer and Burckhardt 1997, Seljak et al. 2008).

Biology. Strictly oligophagous on *Salix* spp. (Salicaceae), *C.nigrita* is reported especially on *S.caprea* L., *S.elaeagnos* Scop., *S.foetida* Schleicher, *S.helvetica* Vill., *S.lapponum* L., *S.myrsinifolia* Salisb., *S.phylicifolia* L., *S.purpurea* L. and *S.waldsteniana* Willd. (Ossiannilsson 1992, Conci et al. 1993, Conci et al. 1996, Ripka 2008, Seljak et al. 2008). It performs only one generation per year and overwinters as an adult on shelter plants (conifers).

***Cacopsyllaperegrina* (Foerster, 1848)**

(Figs 2, 6, 10, 14)

Findings in Luxembourg. Personal field collection by the authors: Oesling: Marnach (11 ♂♂, 17 ♀♀, 2.VI.2020, on *Crataegusmonogyna*), Wahl (2 ♂♂, 2 ♀♀, 14.VIII.2019, on *C.monogyna*); West Gutland: Arsdorf (2 ♀♀, 20.V.2020, on *C.monogyna*), Dondelange (3 ♀♀, 28.V.2020, on *C.monogyna*; 1 ♂, 1 ♀, 28.V.2020, on *Alnusglutinosa*, occasional plant), Elvange/Schweich (7 ♂♂, 7 ♀♀, 28.V.2020, on *C.monogyna*), Esch-sur-Sûre (1 ♀, 15.V.2020, on *C.monogyna*; 1 ♀, 15.V.2020, on *Acer* sp., occasional plant), Strassen (4 ♀♀, 27.IV.2020, on *C.monogyna*), Useldange (2 ♂♂, 2 ♀♀, 28.V.2020, on *C.monogyna*); Minette: Belvaux (9 ♂♂, 2 ♀♀, 8.VIII.2019, on *C.monogyna*; 1 ♂, 8.VIII.2019, on *Euonymus* sp., occasional plant), Dudelange (5 ♂♂, 3 ♀♀, 23.VII.2019, on *C.monogyna*), Kayl (4 ♂♂, 7 ♀♀, 11.VI.2020, on *Malus* sp., occasional plant), Kayl/Tetange (14 ♂♂, 10 ♀♀, 23.VII.2019, on *C.monogyna*), Vesquenhaff (7 ♂♂, 17 ♀♀, 30.VII.2019, on *C.monogyna*); Moselle: Elvange/Burmerange (8 ♂♂, 2 ♀♀, 19.V.2020, on *C.monogyna*), Erpeldange (15 ♂♂, 13 ♀♀, 3 nymphs, 7.V.2020, on *C.monogyna*), Mondorf (4 ♂♂, 3 ♀♀, 18.V.2020, on *C.monogyna*; 2 ♀♀, 18.V.2020, on *Acercampestre*, occasional plant), Moutfort (1 ♀, 1.VI.2020, on *Acerplatanoides*, occasional plant), Remerschen (1 ♀, 28.V.2020, on *Alnusglutinosa*, occasional plant).

Material studied in the MNHNL collection: Oesling: Lellingen, Op Baerel (2 ♂♂, 8.VI - 20.VI.2000, Malaise); East Gutland: Godbrange, Schléidelbierg (7 ♂♂, 4 ♀♀, 18.V - 27.V.1999; 9 ♂♂, 4 ♀♀, 27.V - 9.VI.1999; 14 ♂♂, 10 ♀♀, 9.VI - 25.VI.1999; 1 ♂, 2 ♀♀, 25.VI - 8.VII.1999; 4 ♂♂, 4 ♀♀, 8.VII - 22.VII.1999; 3 ♂♂, 1 ♀, 22.VII - 5.VIII.1999; 2 ♂♂, 5.VIII - 19.VIII.1999; 1 ♀, 19.VIII - 2.IX.1999; 1 ♂, 2 ♀♀, 2.IX - 27.IX.1999, Malaise), Niederanven, Aarnescht (1 ♀, 11.V - 27.V.1999; 2 ♀♀, 27.V - 9.VI.1999; 3 ♂♂, 1 ♀, 9.VI - 25.VI.1999; 1 ♀, 5.VIII - 19.VIII.1999; 2 ♀♀, 2.IX - 27.IX.1999, Malaise).

New record for Luxembourg.

Geographical distribution. Widely distributed in the Palaearctic Region, where it is recorded from Japan and Far East Asia (Inoue 2010) and westwards through the temperate areas of Asia (Burckhardt 2005) and Turkey (Burckhardt and Önuçar 1993), to nearly all Europe and North Africa (Hodkinson and White 1979, Burckhardt 1988a, Ossiannilsson 1992, Conci et al. 1993, Conci et al. 1996, Burckhardt 2005, Seljak 2006, Ripka 2008, Malumphy et al. 2009, Gertsson 2010, Inoue 2010, Lauterer 2011, Malenovský et al. 2011, O’Connor and Malumphy 2011, Kanturski and Drohojowska 2013, Serbina et al. 2015, Drohojowska and Klasa 2019, den Bieman et al. 2019). Recently recorded also from North America (Canada and USA; Wheeler and Stoops 2001, Wheeler and Hoebeke 2005), where it has been introduced.

Biology. Strictly oligophagous on hawthorns (*Crataegus* L., Rosaceae) (Hodkinson and White 1979, Burckhardt 1983, Burckhardt 1988a, Ossiannilsson 1992, Conci et al. 1993, Conci et al. 1996, Burckhardt 2005, Wheeler and Hoebeke 2005, Seljak 2006, Ripka 2008, Hodkinson 2009, Malumphy et al. 2009, Inoue 2010, Lauterer 2011, Malenovský et al. 2011). In Europe, preferably found on *Crataegusmonogyna* Jacq. and *C.oxyacantha* L.; also frequent on other hawthorn species, such as on *C.arnoldiana* Sarg., *C.laevigata* (Poir.) DC or *C.maximowiczii* C.K. Schneid., especially in other geographical areas. On its host plants, this insect performs a single generation per year, with long-living adults during summer, when they frequently spread around also on occasional plants. Eggs are laid in late summer on the host plants (hawthorns) and overwinter. In recent studies, *C.peregrina* showed to be moderately associated with ‘*Candidatus* Liberibacter europaeus’ (Tedeschi et al. 2009, Camerota et al. 2012), a phloem-limited Gram-negative bacterium infecting pear plants though producing no specific symptoms; therefore, thought to be an endophyte rather than a pathogen (Raddadi et al. 2011).

**Cacopsyllasp.gr.pruni (Scopoli, 1763)**

(Figs 2, 6, 10, 14)

Two cryptic species, formerly considered as biotypes A and B, have been evidenced within *C.pruni*. They are ecologically and morphologically indistinguishable and partly overlap in their distributions (Sauvion et al. 2021), but show clear genetic differentiation with no hybrids detected (Sauvion et al. 2007, Peccoud et al. 2013, Peccoud et al. 2018). The identity of the material collected in Luxembourg still needs to be checked by molecular methods.

Findings in Luxembourg. Personal field collection by the authors: West Gutland: Arsdorf (1 ♂, 20.V.2020, on *Prunusspinosa*), Strassen (1 ♀, 27.IV.2020, on *P.spinosa*); Moselle: Elvange/Burmerange (1 ♀, 19.V.2020, on *P.spinosa*).

Material studied in the MNHNL collection: Oesling: Goebelsmühle (1 ♀, 25.V - 8.VI.2000, Malaise), Lellingen, Op Baerel (1 ♀, 25.V - 8.VI.2000; 1 ♀, 8.VI - 20.VI.2000; 2 ♂♂, 1 ♀, 20.VI - 6.VII.2000, Malaise); West Gutland: Capellen, Werwelslach (2 ♂♂, 7 ♀♀, 13.IV - 22.IV.1999; 3 ♀♀, 22.IV - 28.IV.1999; 6 ♂♂, 12 ♀♀, 11.V - 18.V.1999; 1 ♀, 18.V - 27.V.1999; 1 ♂, 1 ♀, 27.V - 9.VI.1999, Malaise); East Gutland: Godbrange, Schléidelbierg (1 ♂, 1 ♀, 6.IV - 22.IV.1999; 2 ♀♀, 11.V - 18.V.1999; 2 ♀♀, 18.V - 27.V.1999, Malaise), Koedange, Poenn (1 ♀, 22.VII - 5.VIII.1999, Malaise), Niederanven, Aarnescht (1 ♂, 1 ♀, 22.IV - 11.V.1999, Malaise); Minette: Schifflange, Kayl, Brucherbierg (1 ♀, 22.IV - 11.V.1999, Malaise); Moselle: Canach, Wéngertsbierg (2 ♂♂, 1 ♀, 22.IV - 11.V.1999; 2 ♂♂, 1 ♀♀, 11.V - 27.V.1999; 1 ♀, 25.VI - 8.VII.1999, 1 ♂, 5.VIII - 19.VIII.1999, Malaise).

New record for Luxembourg.

Geographical distribution. Central Asian-European chorotype; *C.pruni* has been recorded from the Mongolian Region (Irkutsk) westwards to the Iran, Caucasian Region (Azerbaijan, Georgia), Turkey and to nearly all of Europe (Hodkinson and White 1979, Burckhardt 1983, Ossiannilsson 1992, Burckhardt and Lauterer 1993, Conci et al. 1993, Conci et al. 1996, Burckhardt 2005, Seljak 2006, Sauvion et al. 2007, Ripka 2008, Gertsson 2010, Lauterer 2011, Malenovský et al. 2011, O’Connor and Malumphy 2011, Steffek et al. 2012, Kanturski and Drohojowska 2013, Drohojowska and Burckhardt 2014, Ouvrard et al. 2015, Drohojowska and Klasa 2019, den Bieman et al. 2019).

Biology. Though its adults have been found by chance also on occasional plants, such as *Crataegus* spp. or *Malusdomestica* Borckh. (Oettl and Schlink 2015), *C.pruni* is strictly oligophagous on plants of the genus *Prunus* L. (Rosaceae), with *Prunusspinosa* L. being the most frequent host plant, but it can also be observed on *P.armeniaca* L., *P.avium* (L.) L., *P.cerasifera* Ehrh., *P.domestica* L., *P.insititia* L., *P.padus* L., *P.persica* (L.) Stokes and *P.salicina* Lindl. (Hodkinson and White 1979, Burckhardt 1983, Ossiannilsson 1992, Conci et al. 1993, Conci et al. 1996, Burckhardt 2005, Seljak 2006, Sauvion et al. 2007, Ripka 2008, Hodkinson 2009, Lauterer 2011, Malenovský et al. 2011, Steffek et al. 2012, Jarausch and Jarausch 2016). This psyllid species performs one generation per year and overwinters as an adult on shelter plants (conifers). Recent studies allowed us to understand the feeding behaviour on its winter shelter plants, through the application of electrical penetration graph (EPG) recordings and survival bioassays on different conifer species, as well as the analysis of chemical composition of their plant sap (Gallinger and Gross 2018).

Economic significance. Over the past few years, *C.pruni* has taken on a remarkable phytosanitary significance, for its ability to transmit '*Candidatus* Phytoplasma prunorum' (Carraro et al. 1998, Jarausch et al. 2001, Carraro et al. 2004), the causing agent of a complex of economically important disorders on *Prunus* plants (including cultivated *P.armeniaca*, *P.domestica* and *P.persica*), which are collectively referred to as European Stone Fruit Yellows (ESFY). Recently, it has been demonstrated how both putative species of the *C.pruni* complex can transmit and spread the pathogen (Marie-Jeanne et al. 2020). Over the past two decades, ESFY has been detected in most southern and central European countries, Middle East and North Africa (Sertkaya et al. 2005, Marcone et al. 2010, Ben Khalifa et al. 2011, Cieślińska 2011, Tedeschi et al. 2013, Allahverdi et al. 2014, Valasevich and Schneider 2016, Warabieda et al. 2017, Andrianjaka-Camps et al. 2018, Jarausch et al. 2019, Riedle-Bauer et al. 2019b), thus being one of the most serious pests in European stone fruit production and a potential threat for fruit crops worldwide, causing important economic damage.

Given its phytosanitary importance as a potential vector of serious phytopathogenic microorganisms, it would be worth monitoring the distribution and pathogenicity of the separate taxonomic entities within the *Cacopsyllapruni* complex in Luxembourg.

***Cacopsyllapulchra* (Zetterstedt, 1838)**

(Figs 2, 6, 10, 14)

Findings in Luxembourg. Personal field collection by the authors: East Gutland: Rodenburg (1 ♂, 2 ♀♀, 27.V.2020, on *Salixcaprea*); Moselle: Mondorf (1 ♂, 2 ♀♀, 18.V.2020, on *Salix* sp.).

Material studied in the MNHNL collection: Minette: Kockelscheuer, Conter Jans Boesch (1 ♀, 13.IV - 22.IV.1999, Malaise), Schifflange, Kayl, Brucherbierg (1 ♂, 11.V - 27.V.1999, Malaise).

New record for Luxembourg.

Geographical distribution. *Cacopsyllapulchra* is a Eurasian chorotype, widely distributed in Europe (except the southern Balkan Peninsula) and recorded also from the Caucasian Region and eastwards to Central (Mongolia) and East Asia (Far East Russia, Japan, Korean Peninsula) (Hodkinson and White 1979, Kwon 1983, Ossiannilsson 1992, Conci et al. 1993, Conci et al. 1996, Lauterer and Burckhardt 1997, Seljak 2006, Ripka 2008, Gertsson 2010, Inoue 2010, Malenovský et al. 2011, O’Connor and Malumphy 2011, Ouvrard et al. 2015, Serbina et al. 2015, Drohojowska and Klasa 2019, den Bieman et al. 2019).

Biology. Strictly oligophagous on *Salix* spp. (Salicaceae), *C.pulchra* has been recorded on many species of willows, such as *S.apennina* Skvortsov, *S.atrocinerea* Brot., *S.caprea* L., *S.cinerea* L., *S.elaeagnos* Scop., *S.foetida* Schleicher, *S.gracilistyla* Miq., *S.integra* Thunb., *S.myrsinifolia* Salisb., *S.pentandra* L., *S.purpurea* L., *S.repens* L. and *S.viminalis* L. (Hodkinson and White 1979, Burckhardt 1983, Kwon 1983, Ossiannilsson 1992, Conci et al. 1993, Conci et al. 1996, Park 1996, Seljak 2006, Malenovský et al. 2011, Cho et al. 2017a). It performs one generation per year and overwinters as an adult on shelter plants (conifers).

***Cacopsyllapyri* (Linnaeus, 1758)**

(Figs 2, 6, 10, 14)

Findings in Luxembourg. Personal field collection by the authors: Moselle: Burmerange (6 ♂♂, 8 ♀♀, 8.V.2020, on *Pyruscommunis*), Elvange/Burmerange (2 ♂♂, 3 ♀♀, 19.V.2020, on *P.communis*).

New record for Luxembourg.

Geographical distribution. *Cacopsyllapyri* is common throughout Europe, especially in the central-southern fruit growing areas of the continent and is recorded also from the Caucasian Region (Armenia, Georgia), Middle East (Iran), central Asia (Kazakhstan) eastwards up to China (Xinjiang) (Hodkinson and White 1979, Burckhardt 1983, Burckhardt 1988a, Ossiannilsson 1992, Conci et al. 1993, Burckhardt and Lauterer 1993, Burckhardt and Önuçar 1993, Conci et al. 1996, Aguiar and Martin 2001, Agusti et al. 2003, Seljak 2006, Ripka 2008, O’Connor and Malumphy 2011, Sanchez and Ortin-Angulo 2011, Sanchez and Ortin-Angulo 2012, Drohojowska and Burckhardt 2014, Bufaur and Harizanova 2015, Oettl and Schlink 2015, Pekár et al. 2015, Serbina et al. 2015, Cho et al. 2017b, Drohojowska and Klasa 2019, den Bieman et al. 2019).

Biology. Oligophagous on plants of the genus *Pyrus* L., mostly *Pyruscommunis* L. (Rosaceae) (Hodkinson and White 1979, Burckhardt 1983, Burckhardt and Hodkinson 1986, Burckhardt 1988a, Ossiannilsson 1992, Conci et al. 1993, Burckhardt 1994a, Conci et al. 1996, Aguiar and Martin 2001, Seljak 2006, Ripka 2008, Hodkinson 2009, Li 2011, Sanchez and Ortin-Angulo 2011, Civolani et al. 2013). According to Cravedi et al. (2008), it performs up to 5-7 generations per year, overwintering as adults (especially females in ovarian diapause) in bark crevices of the same pear trees, but also on other occasional plants. Egg deployment starts in early spring, with the first sunny days (2-3 consecutive days with maximum temperatures over 10°C), initially within the cracks of the twigs, then in those of smaller branches and afterwards, as soon as trees start to vegetate, on perules, petioles and leaflets. Newly-emerged nymphs tend to settle during this spring period on the newly-spread leaflets and around the calycin zone of the newly-formed small fruits. Later in the season, adults of the summer generations lay eggs on tender shoots, along the main vein of the underside of the leaves, where nymphs tend to remain and produce a huge amount of honeydew. Late summer and autumn generations have normally a reduced fertility; and population levels are usually reduced with consequent limited harm, although late summer pullulations may also occur (especially in warmer climates).

Economic significance. *Cacopsyllapyri* is the most common psyllid species on all cultivated pear varieties in western Europe and regionally often considered a key pest. Nymphs of the summer generation produce a large amount of honeydew, damaging both plants and fruits. In the case of heavy infestations, the honeydew can smear all green parts of the plant, reducing photosynthesis and respiration, as well as making fruits suffer a significant commercial depreciation. *Cacopsyllapyri* can also be dangerous, especially to young plants, as a vector of ‘*Candidatus* Phytoplasma pyri’, the agent of Pear Decline disease, which is transmitted in Europe by both nymphs and adults of this psyllid (Facundo et al. 2017, Riedle-Bauer et al. 2019a). Transmission from one year to the next can be sustained by infected overwintering adult specimens.

Numerous species of entomophagous insects are known as natural antagonists of *C.pyri*, both parasitoids, such as *Prionomitusmitratus* (Dalman) or *Trechnitespsyllae* (Ruschka) and predators, such as species of the genera *Anthocoris* Fallen and *Orius* Wolff (Hemiptera, Anthocoridae). In particular, the predation by *Anthocorisnemoralis* (Fabr.) is highly significant; and the integrated control of this pear psyllid must be primarily based on techniques aimed at protecting and favouring (through augmentative releases) the action of this natural antagonist. This is why, in case of strong infestation requiring chemical control, low impact applications with selective insecticides having deterrent effects on oviposition (such as plant oils) (Erler and Tosun 2017) or with products having washing properties on the honeydew (such as salts of fatty acids) can be applied in mid-spring against the second generation nymphs, if intervention thresholds are exceeded.

In order to reduce the incidence of the Pear Decline phytoplasma, especially on young plants, control should be directed against overwintering adults of *C.pyri*, with the main objective of reducing the vector population before the spring vegetation of plants, thus preventing the transmission on time.

***Cacopsyllapyricola* (Foerster, 1848)**

(Figs 2, 6, 10, 14)

Findings in Luxembourg. Personal field collection by the authors: West Gutland: Esch-sur-Sûre (1 ♂, 3 ♀♀, 15.V.2020, on *Pyruscommunis*), Noerdange (1 ♂, 1 ♀, 28.V.2020, on *P.communis*); East Gutland: Bettendorf (3 ♂♂, 6 ♀♀, 27.V.2020, on *P.communis*), Olingen (6 ♂♂, 6 ♀♀, 27.V.2020, on *P.communis*); Moselle: Burmerange (1 ♀, 8.V.2020, on *P.communis*), Elvange (1 ♀, 1.VIII.2019, on *P.communis*), Elvange/Burmerange (2 ♀♀, 19.V.2020, on *P.communis*).

Material studied in the MNHNL collection: Minette: Schifflange, Brucherbierg (2 ♂♂, 22.IV - 11.V.1999, Malaise).

New record for Luxembourg.

Geographical distribution. Before the revisions of Burckhardt and Hodkinson (1986) and Cho et al. (2017b), respectively, of the west and east Palaearctic pear psyllids, *C.pyricola* was reported from the Asiatic Far East, Caucasus and all of Europe; and was also indicated as introduced in North and South America. At present, its occurrence is confirmed only in Europe (including the Caucasian Region) and North America (Burckhardt 1988a, Hodkinson 1988, Ossiannilsson 1992, Burckhardt and Lauterer 1993, Conci et al. 1993, Burckhardt 1994a, Conci et al. 1996, Agusti et al. 2003, Seljak 2006, Ripka 2008, Gertsson 2010, Inoue 2010, O’Connor and Malumphy 2011, Percy et al. 2012, Kanturski and Drohojowska 2013, Cooper and Horton 2015, Castillo Carrillo et al. 2016, Zendedel et al. 2016, Cho et al. 2017b, Holzinger et al. 2017, Drohojowska and Klasa 2019, den Bieman et al. 2019). As for all other European pear psyllids, the presence of *C.pyri* in East Asia was also questioned, based on recent DNA barcoding studies (Cho et al. 2020).

Biology. Oligophagous on plants of the genus *Pyrus* L. (Rosaceae), especially on *P.communis* L., but also frequently recorded on *P.calleryana* Decne., *P.pyrasater* (L.) and *P.ussuriensis* Maxim. (Burckhardt 1983, Burckhardt 1988a, Hodkinson 1988, Ossiannilsson 1992, Conci et al. 1993, Burckhardt 1994a, Conci et al. 1996, Seljak 2006, Ripka 2008, Hodkinson 2009, Guédot et al. 2009, Inoue 2010, Percy et al. 2012, Cooper and Horton 2015, Spodek et al. 2017). It performs 3-5 generations per year, overwintering as adults on the host plants (but also on other occasional fruit or forestry plants in the surrounding area). In early spring, eggs are laid in groups on the buds; later in the season and throughout all summer, they are mostly laid on the underside of the leaves, along the middle vein or in the grooves of the leaf stalks.

Economic significance. In Europe, *C.pyricola* is the second most important and widespread species of pear psyllids, after *C.pyri*, to which we refer for information on harmfulness and control methods. Apart from the negative effects on infested plants consisting in the suction of sap and production of abundant honeydew, *C.pyricola* is also a vector of the pathogenic microorganisms causing Pear Decline disease (‘*Candidatus* Phytoplasma pyri’) (Facundo et al. 2017, Cruz et al. 2018, Riedle-Bauer et al. 2019a); for such damage, it is particularly feared in North America.

***Cacopsyllapyrisuga* (Foerster, 1848)**

(Figs 2, 6, 10, 14)

Findings in Luxembourg. Personal field collection by the authors: West Gutland: Esch-sur-Sûre (1 ♂, 15.V.2020, on *Pyruscommunis*), Noerdange (1 ♂, 28.V.2020, on *P.communis*); Moselle: Burmerange (1 ♀, 8.V.2020, on *P.communis*).

New record for Luxembourg.

Geographical distribution. Eurasian chorotype, widely distributed in Europe and diffused eastwards, through Turkey, the Middle East, the Caucasian Region and Iran (Ramírez Gómez 1956, Hodkinson and White 1979, Burckhardt 1983, Kwon 1983, Burckhardt 1988a, Ossiannilsson 1992, Conci et al. 1993, Burckhardt and Lauterer 1993, Burckhardt and Önuçar 1993, Conci et al. 1996, Zeidan-Gèze and Burckhardt 1998, Lauterer 1999, Seljak 2006, Ripka 2008, Inoue 2010, Malenovský et al. 2011, Jerinić-Prodanović and Protić 2013, Kanturski and Drohojowska 2013, Serbina et al. 2015, Spodek et al. 2017, Drohojowska and Klasa 2019, den Bieman et al. 2019), though findings in the extreme eastern part of Asia have been recently referred to other taxa (Cho et al. 2017b, Cho et al. 2020).

Biology. Strictly oligophagous on *Pyrus* spp. (Rosaceae), with records in Europe especially on *P.amygdalformis* Vill., *P.communis* L. and *P.elaeagnifolia* Pall.; in Asia, also reported from many other *Pyrus* species, amongst which *P.pashia* Buch.-Ham. ex D. Don, *Pyruspyrifolia* (Burm. f.) Nakai, *P.salicifolia* Pall. and *P.ussurensis* Maxim. ex Rupr. (Hodkinson and White 1979, Burckhardt 1983, Kwon 1983, Burckhardt 1988a, Ossiannilsson 1992, Conci et al. 1993, Burckhardt 1994a, Conci et al. 1996, Zeidan-Gèze and Burckhardt 1998, Seljak 2006, Ripka 2008, Hodkinson 2009, Inoue 2010, Malenovský et al. 2011). It performs one generation per year and overwinters as an adult on shelter plants (conifers). Sometimes, it causes deformations to leaves, which appear wrinkled and folded (Houard 1908, Houard 1909, Houard 1913).

Economic significance. In the past, *C.pyrisuga* has been reported to be harmful to pears. Current knowledge suggests that it does not cause direct damage, being apparently better adapted to wild pears or old varieties of cultivated pear trees, especially in hilly areas and in non-intensive orchards. However, it has been recently confirmed as a vector of "*Ca.* Phytoplasma pyri", the causing agent of Pear Decline (Riedle-Bauer et al. 2022).

***Cacopsyllarhamnicola* (Scott, 1876)**

(Figs 2, 6, 10)

Findings in Luxembourg. Personal field collection by the authors: West Gutland: Brouch (3 ♀♀, 15.V.2020, on *Salixcaprea*, occasional plant), Strassen (2 ♀♀, 27.IV.2020; 1 ♀, 8.VI.2020, by general sweeping with net).

New record for Luxembourg.

Geographical distribution. Widely distributed from central Asia to nearly all Europe (Hodkinson and White 1979, Burckhardt 1988b, Ossiannilsson 1992, Burckhardt and Önuçar 1993, Conci et al. 1993, Conci et al. 1996, Seljak 2006, Ripka 2008, Ripka 2009, Gertsson 2010, Malenovský et al. 2011, O’Connor and Malumphy 2011, Ouvrard et al. 2015, den Bieman et al. 2019).

Biology. Strictly oligophagous on *Rhamnus* spp. (Rhamnaceae), especially *R.cathartica* L., but also reported on *R.alpinus* L., *R.fallax* Boiss., *R.imeretina* Booth et al. and *R.saxatilis* Jacq. (Hodkinson and White 1979, Burckhardt 1983, Burckhardt 1988b, Ossiannilsson 1992, Conci et al. 1993, Conci et al. 1996, Seljak 2006, Ripka 2008, Ripka 2009, Hodkinson 2009, Malenovský et al. 2011); records in Luxembourg refer only to occasional plants, so far. *Cacopsyllarhamnicola* performs only one generation per year and overwinters as an adult on shelter plants (conifers).

***Cacopsyllaulmi* (Foerster, 1848)**

Findings in Luxembourg. Personal field collection by the authors: Moselle: Elvange/Burmerange, (2 ♂♂, 1 ♀, 1.VI.2020, by sweeping with net on a mix with *Acerplatanoides* + *Populuscinerea*, occasional plants).

New record for Luxembourg.

Geographical distribution. *Cacopsyllaulmi* is a Eurasian chorotype, occurring in most parts of central and northern Europe, the Caucasian Region and central Asia (Hodkinson and White 1979, Burckhardt 1983, Ossiannilsson 1992, Conci et al. 1993, Conci et al. 1996, Hansen 1996, Seljak 2006, Ripka 2008, Lauterer 2011, Malenovský et al. 2011, Kanturski and Drohojowska 2013, Serbina et al. 2015, Drohojowska and Klasa 2019, den Bieman et al. 2019).

Biology. Strictly oligophagous on elm trees (*Ulmus* spp., Ulmaceae), *C.ulmi* has been especially found on *Ulmuseffusa* Willd., *U.glabra* Huds., *U.laevis* Pall., *U.minor* Mill. and *U.pedunculata* Foug. (Hodkinson and White 1979, Burckhardt 1983, Ossiannilsson 1992, Conci et al. 1993, Conci et al. 1996, Hansen 1996, Seljak 2006, Ripka 2008, Hodkinson 2009, Lauterer 2011, Malenovský et al. 2011). It performs one generation per year and spends the winter as an egg on the host plants. According to Lauterer (1991), part of its population migrates during the summer months to other plant species (mainly trees), which are used for shelter.

***Cacopsyllavisci* (Curtis, 1835)**

(Figs 2, 6, 10)

Findings in Luxembourg. Personal field collection by the authors: Moselle: Elvange/Burmerange (5 ♀♀, 19.V.2020, on *Viscumalbum*).

New record for Luxembourg.

Geographical distribution. *Cacopsyllavisci* is likely a Palaearctic chorotype. It is reported from nearly all of Europe (Hodkinson and White 1979, Lauterer 1979, Conci et al. 1993, Conci et al. 1996, Hansen and Hodkinson 2006, Seljak 2006, Ripka 2008, Varga et al. 2012, Ouvrard et al. 2015, Burckhardt et al. 2017, Struwe et al. 2009, Drohojowska and Klasa 2019, den Bieman et al. 2019), from North Africa (Morocco) (Inoue 2010), the Middle East and the Caucasian Region (Inoue 2010, Burckhardt et al. 2017), to Far East Asia (Japan and South Korea) (Inoue 2010, Cho et al. 2017a).

Biology. *Cacopsyllavisci* lives only on Santalales of the genera *Loranthus* Jacq. (*L.europaeus* Jacq.) (Loranthaceae) and *Viscum* L. (*V.album* L., *V.laxum* Boiss. & Reut.) (Santalaceae) (Hodkinson and White 1979, Burckhardt 1983, Hansen and Hodkinson 2006, Seljak 2006, Hodkinson 2009, Inoue 2010). According to Bin (1970), it performs 2-3 generations per year, overwintering in the nymphal stage on its host plants, to which it causes showy deformations to the leaves, which are folded in a C-shape.

***Livillaulicis* Curtis, 1836**

(Figs 3, 7, 11, 15)

Findings in Luxembourg. Material studied in the MNHNL collection: East Gutland: Niederanven, Aarnescht (4 ♂♂, 3 ♀♀, 27.V - 9.VI.1999; 1 ♀, 9.VI - 25.VI.1999, Malaise).

New record for Luxembourg.

Geographical distribution. Central European chorotype, never recorded till now in the northernmost part of the continent (Scandinavia), as well as in the “Benelux” Region and the Iberian Peninsula (Hodkinson and White 1979, Burckhardt 1983, Hodkinson and Hollis 1987, Conci et al. 1993, Conci et al. 1996, Seljak 2006, O’Connor and Malumphy 2011).

Biology. Oligophagous on plants of the genus *Genista* L. (Fabaceae); especially recorded from *G.tinctoria* L. and *G.germanica* L. (Hodkinson and White 1979, Burckhardt 1983, Hodkinson and Hollis 1987, Conci et al. 1993, Conci et al. 1996, Seljak 2006); records on other Fabaceae, such as *Cytisusscoparius* (L.) Link or *Ulexeuropaeus* L., are not confirmed. *Livillaulicis* performs one generation per year and probably spends the winter in the egg stage on its host plants.

***Psyllaalni* (Linnaeus, 1758)**

(Figs 3, 7, 11, 15)

Findings in Luxembourg. Personal field collection by the authors: West Gutland: Dondelange (5 ♂♂, 2 ♀♀, 28.V.2020, on *Alnusglutinosa*; 1 ♂, 28.V.2020, on *Crataegusmonogyna*, occasional plant), Elvange/Schweich (1 ♂, 28.V.2020, on *Prunusspinosa*, occasional plant), Goesdorf/Bockholtz (7 ♂♂, 4 ♀♀, 20.V.2020, on *A.glutinosa*), Hobscheid (1 ♂, 28.V.2020, on *A.glutinosa*), Préitzerdaul (7 ♂♂, 5 ♀♀, 20.V.2020, on *A.glutinosa*); East Gutland: Bettendorf (2 ♂♂, 2 ♀♀, 27.V.2020, on *A.glutinosa*), Rodenburg (3 ♂♂, 4 ♀♀, 27.V.2020, on *A.glutinosa*); Minette: Belvaux (1 ♂, 1 ♀, 19.VII.2019, on *A.glutinosa*), Kayl (1 ♂, 20.V.2020, on *Malus* sp., occasional plant).

New record for Luxembourg.

Geographical distribution. Distributed in the Holarctic Region, with a wide presence in the Palaearctic (though not reported from North Africa) and in North America (Canada, Greenland, USA) (Hodkinson and White 1979, Kwon 1983, Yang 1984, Hodkinson 1986, Hodkinson 1988, Ossiannilsson 1992, Conci et al. 1993, Burckhardt and Önuçar 1993, Conci et al. 1996, Labina 2006, Seljak 2006, Ripka 2008, Gertsson 2010, Inoue 2010, Malenovský et al. 2011, O’Connor and Malumphy 2011, Percy et al. 2012, Kanturski and Drohojowska 2013, Drohojowska and Burckhardt 2014, Ouvrard et al. 2015, Serbina et al. 2015, Holzinger et al. 2017, Drohojowska and Klasa 2019, den Bieman et al. 2019).

Biology. Strictly oligophagous on various species of alders (*Alnus* Mill., Betulaceae). In Europe, on *Alnusglutinosa* (L.) Gaertn. and *A.incana* (L.) Moench and sometimes on *A.viridis* (Chaix) DC.; in Asia, also on *A.hirsuta* Turcz. ex Rupr. and *A.japonica* (Thunb.) Steud.; in North America, on *A.rhombifolia* Nutt. (Hodkinson and White 1979, Yang 1984, Hodkinson 1986, Ossiannilsson 1992, Conci et al. 1993, Conci et al. 1996, Seljak 2006, Ripka 2008, Inoue 2010, Percy et al. 2012). On its host plants, this psyllid species performs only one generation per year, overwintering as an egg which is laid inside the shoots.

***Psyllabetulae* (Linnaeus, 1758)**

(Figs 3, 7, 11, 15)

Findings in Luxembourg. Personal field collection by the authors: Moselle: Remerschen (3 ♂♂, 4 ♀♀, 28.V.2020, on *Betulapendula*).

New record for Luxembourg.

Geographical distribution. *Psyllabetulae* is a Holarctic chorotype, widely distributed in central and northern Europe, but recorded also from many parts of Asia (Georgia, Mongolia, Far East Russia, Japan) and North America (Canada) (Hodkinson and White 1979, Hodkinson and MacLean 1980, Hodkinson 1988, Ossiannilsson 1992, Conci et al. 1993, Conci et al. 1996, Labina 2006, Labina 2008, Gertsson 2010, Inoue 2010, O’Connor and Malumphy 2011, Serbina et al. 2015).

Biology. Strictly oligophagous on birches (*Betula* spp., Betulaceae), according to literature, it lives in Europe mainly on *B.pendula* Roth. or *B.pubescens* Ehrh. (Hodkinson and White 1979, Ossiannilsson 1992, Conci et al. 1993, Conci et al. 1996); recorded in Asia also on many different *Betula* species, such as *B.middendorfii* Trautvetter & C.A. Meyer, *B.ermanii* Cham., *B.platyphylla* Sukaczev and others (Hodkinson and MacLean 1980, Inoue 2010). It performs one generation per year and overwinters as eggs on its host plants.

***Psyllafoersteri* Flor, 1861**

(Figs 3, 7, 11, 15)

Findings in Luxembourg. Personal field collection by the authors: Minette: Belvaux (2 ♂♂, 6 ♀♀, 19.VII.2019, on *Alnusglutinosa*); Moselle: Remerschen (3 ♂♂, 4 ♀♀, 28.V.2020, on *A.glutinosa*).

Material studied in the MNHNL collection: Oesling: Sonlez, Pamer (1 ♂, 24.VIII - 22.IX.2000, Malaise).

New record for Luxembourg.

Geographical distribution. The occurrence of *P.foersteri* is confirmed in the West Palaearctic Region, where it is present in most parts of Europe, North Africa (Algeria) and the Middle East (Caucasian Region, Lebanon, Turkey) (Hodkinson and White 1979, Burckhardt 1983, Burckhardt 1988b, Burckhardt 1989, Ossiannilsson 1992, Conci et al. 1993, Burckhardt and Önuçar 1993, Conci et al. 1996, Zeidan-Gèze and Burckhardt 1998, Seljak 2006, Ripka 2008, Gertsson 2010, O’Connor and Malumphy 2011, Kanturski and Drohojowska 2013, Ouvrard et al. 2015, Serbina et al. 2015, den Bieman et al. 2019). Recorded also from New Zealand (Martoni et al. 2018), where it has been introduced.

Biology. Oligophagous on alder trees (*Alnus* Mill., Betulaceae); especially recorded on *Alnusglutinosa* (L.) Gaertn. and *A.incana* (L.) Moench (Hodkinson and White 1979, Ossiannilsson 1992, Conci et al. 1993, Conci et al. 1996, Zeidan-Gèze and Burckhardt 1998, Seljak 2006, Ripka 2008). Performs only one generation per year and overwinters in the egg stage on its host plants.

***Psyllahartigii* Flor, 1861**

(Figs 3, 7, 11, 15)

Findings in Luxembourg. Personal field collection by the authors: West Gutland: Strassen (4 ♂♂, 2 ♀♀, 27.IV.2020, on *Betulapendula*); Minette: Belvaux (2 ♂♂, 7.V.2020, on *B.pendula*; 1 ♀, 8.V.2020, on *B.pendula*).

New record for Luxembourg.

Geographical distribution. It probably has a wide distribution in the Holarctic Region; in addition to larger parts of Europe (especially its central and northern areas) (Hodkinson and White 1979, Ossiannilsson 1992, Conci et al. 1993, Conci et al. 1996, Seljak 2006, Ripka 2008, Gertsson 2010, O’Connor and Malumphy 2011, Kanturski and Drohojowska 2013, Serbina et al. 2015, Drohojowska and Klasa 2019, den Bieman et al. 2019), reports of *C.hartigii* concern the Far East (Japan and other nearby islands, such as Kuril and Sakhalin) and central Asia (Altai Province in Russia), the Caucasian Region and North America (USA and Canada) (Hodkinson 1988, Ossiannilsson 1992, Labina 2008, Inoue 2010).

Biology. Strictly oligophagous on birches (genus *Betula* L., Betulaceae), it has been found in Europe on *Betulaalba* L., *B.pendula* Roth. and *B.pubescens* Ehrh., but records also originate from *B.platyphylla* Sukaczev and *B.raddeana* Trautv. in Asia, as well as *B.populifolia* Marshall in North America (Hodkinson and White 1979, Hodkinson 1988, Ossiannilsson 1992, Conci et al. 1993, Conci et al. 1996, Inoue 2010). It shows only one generation per year and overwinters in the egg (or perhaps in the nymphal) stage (Lauterer 1976).

***Spanioneurabuxi* (Linnaeus, 1758)**

(Figs 3, 7, 11, 15)

Findings in Luxembourg. Galls produced by this psyllid on *Buxussempervirens* are reported by Lambinon and Schneider (2004) (Oesling: Clervaux; Minette: Dudelange, Esch-sur-Alzette Mosel: Palmberg, Ahn; West Gutland: Bonnevoie, Bridel, Capellen; East Gutland: Diekirch, Ettelbruck, Stackels, Galgebierg, Betzdorf). Explicit mention on its finding in Luxembourg is provided by Eickermann et al. (2020).

Personal field collection by the authors: Oesling: Clervaux (1 ♀, 12.IX.2019, on *B.sempervirens*), Hosingen (1 ♀, 12.IX.2019, on *B.sempervirens*), Vianden (2 ♂♂, 4 ♀♀, 12.IX.2019, on *B.sempervirens*); West Gutland: Bettborn (3 ♀♀, 12.IX.2019, on *B.sempervirens*), Roodt-sur-Eisch (2 ♂♂, 5 ♀♀, 22.VIII.2019, on *B.sempervirens*), Strassen (4 ♂♂, 5 ♀♀, 22.VIII.2019, on *B.sempervirens*); East Gutland: Niederfeulen (1 ♂, 5 ♀♀, 12.IX.2019, on *B.sempervirens*), Reisdorf (1 ♂, 1 ♀, 12.IX.2019, on *B.sempervirens*); Moselle: Elvange/Burmerange (1 ♂, 1.VI.2020, by sweeping with net on a mix with *Acerplatanoides* + *Populuscinerea*, occasional plants). Galls produced by *P.buxi* on *B.sempervirens* have been also detected by us in Weiler (Oesling), Bereldange, Colmar-Berg, Mersch and Steinsel (West Gutland), Luxembourg Ville (Minette).

Geographical distribution. Widely diffused in Europe, from where it seems to be native (Hodkinson and White 1979, Ossiannilsson 1992, Conci et al. 1993, Conci et al. 1996, Seljak 2006, Ripka 2008, O’Connor and Malumphy 2011, Kanturski and Drohojowska 2013, Ouvrard et al. 2015, Serbina et al. 2015, den Bieman et al. 2019, Eickermann et al. 2020. Reported also from the Nearctic Region (Canada, USA, including Hawaii), where it has been probably introduced (Wheeler and Hoebeke 2005, Percy et al. 2012, Castillo Carrillo et al. 2016).

Biology. Strictly oligophagous on *Buxus* spp. (Buxaceae), this psyllid species has been reported on *Buxusbalearica* Lam., *B.macrophylla* (Britton) Fawc. & Rendle and *B.sempervirens* L. (Hodkinson and White 1979, Ossiannilsson 1992, Conci et al. 1993, Conci et al. 1996, Burckhardt 2005, Percy et al. 2012). It performs one generation per year, always remaining on the host plants, on which it overwinters in the egg stage (or even as first instar nymph, according to environmental conditions) (Eickermann et al. 2020). *P.buxi* causes singular galls to box plants, creating “spoon-like” folds of the apical leaves, which are often close together for the shortening of the internodes, generating the typical "artichoke" appearance of the most complex galls.

***Spanioneurafonscolombii* Foerster, 1848**

(Figs 3, 7, 11, 15)

Findings in Luxembourg. First reported from Luxembourg by O’Connor and Malumphy (2011).

Personal field collection by the authors: Oesling: Clervaux (6 ♂♂, 8 ♀♀, 12.IX.2019, on *Buxussempervirens*), Hosingen (1 ♀, 12.IX.2019, on *B.sempervirens*), Vianden (1 ♂, 2 ♀♀, 12.IX.2019, on *B.sempervirens*), Weller (6 ♂♂, 1 ♀, 12.IX.2019, on *B.sempervirens*); West Gutland: Roodt-sur-Eisch (20 ♂♂, 20 ♀♀, 22.VIII.2019, on *B.sempervirens*); East Gutland: Niederfeulen (5 ♂♂, 4 ♀♀, 12.IX.2019, on *B.sempervirens*), Reisdorf (5 ♂♂, 12 ♀♀, 12.IX.2019, on *B.sempervirens*).

Geographical distribution. As far as presently inferred from literature, *S.fonscolombii* has a predominantly western European distribution, with records originating from Belgium, France, Great Britain, Ireland, Italy, Luxembourg, Slovenia, Spain, Sweden and Switzerland (Hodkinson and White 1979, Conci et al. 1993, Conci et al. 1996, Burckhardt and Mühlethaler 2003, Seljak 2006, Gertsson 2010, O’Connor and Malumphy 2011, Ouvrard et al. 2015, den Bieman et al. 2019); it is also reported from the Caucasian Region (Azerbaijan; Conci et al. 1996) and from USA (Hodkinson 1988), where it has been probably introduced.

Biology. Strictly oligophagous on *Buxus* spp., especially found on *B.sempervirens* L. (Buxaceae) (Hodkinson and White 1979, Burckhardt 1983, Hodkinson 1988, Conci et al. 1993, Conci et al. 1996, Burckhardt and Mühlethaler 2003, Seljak 2006, Hodkinson 2009). It performs one generation per year and overwinters in the adult stage on its host plants.

### Triozidae

***Bactericeraalbiventris* (Foerster, 1848)**

(Figs 4, 8, 12)

Findings in Luxembourg. Personal field collection by the authors: Minette: Belvaux (2 ♀♀, 19.VII.2019, on *Salixalba*; 1 ♀, 8.VIII.2019, on *S.alba*; 1 ♀♀, 8.VIII.2019, on *Crataegusmonogyna*, occasional plant).

New record for Luxembourg.

Geographical distribution. *Bactericeraalbiventris* is an Eurasian chorotype, widely distributed in nearly all of Europe and eastwards to the Middle East (Iran, Israel, Lebanon, Turkey), Central Asia (Afghanistan, Caucasian Region, Mongolia, Tadzhikistan, Turkmenistan, Uzbekistan) to East Asia (Far East Russia) (Hodkinson and White 1979, Burckhardt 1988a, Burckhardt 1988b, Halperin et al. 1988, Ossiannilsson 1992, Burckhardt and Önuçar 1993, Conci et al. 1996, Burckhardt and Lauterer 1997a, Zeidan-Gèze and Burckhardt 1998, Burckhardt 2005, Seljak 2006, Ripka 2008, Hodkinson 2009, Gertsson 2010, O’Connor and Malumphy 2011, Malenovský et al. 2012, Kanturski and Drohojowska 2013, Zendedel et al. 2016, Spodek et al. 2017, Drohojowska and Klasa 2019, den Bieman et al. 2019).

Biology. Strictly oligophagous on *Salix* spp. (Salicaceae), with records reported in literature on many species of willows, as *S.acmophylla* Boiss., *S.alba* L., *S.amygdalina* L., *S.aurita* L., *S.babylonica* L., *S.elaeagnos* Scop., *S.fragilis* L., *S.pentandra* L., *S.purpurea* L., *S.triandra* L. and *S.viminalis* L. (Hodkinson and White 1979, Burckhardt 1988a, Burckhardt 1988b, Ossiannilsson 1992, Conci et al. 1996, Burckhardt and Lauterer 1997a, Zeidan-Gèze and Burckhardt 1998, Burckhardt 2005, Seljak 2006, Ripka 2008, Hodkinson 2009, Malenovský et al. 2012, Spodek et al. 2017). It performs one or two generations per year and overwinters as an adult on shelter plants (conifers).

***Bactericeracurvatinervis* (Foerster, 1848)**

(Figs 4, 8, 12)

Findings in Luxembourg. Personal field collection by the authors: West Gutland: Brouch (4 ♀♀, 15.V.2020, on *Salixviminalis*).

New record for Luxembourg.

Geographical distribution. Known from most of central and northern Europe, it is reported also from Lebanon, the Caucasus and far east Asia (Ossiannilsson 1992, Conci et al. 1993, Conci et al. 1996, Burckhardt and Lauterer 1997a, Zeidan-Gèze and Burckhardt 1998, Seljak 2006, Ripka 2008, Labina and Evdogarova 2009, Gertsson 2010, O’Connor and Malumphy 2011, Ouvrard et al. 2015, Serbina et al. 2015, den Bieman et al. 2019).

Biology. Strictly oligophagous on willows (*Salix* spp., Salicaceae), reported in literature from *S.alba* L., *S.appendiculata* Vill., *S.aurita* L., *S.caprea* L., *S.cinerea* L., *S.elaeagnos* Scop., *S.glabra* Scop., *S.purpurea* L., *S.repens* L. and *S.viminalis* L. (Ossiannilsson 1992, Conci et al. 1993, Conci et al. 1996, Burckhardt and Lauterer 1997a, Ripka 2008, Labina and Evdogarova 2009). *Bactericeracurvatinervis* performs only one generation per year and overwinters as an adult on shelter plants (conifers).

***Bactericerasubstriola* Ossiannilsson, 1992**

(Figs 4, 8, 12, 16)

Findings in Luxembourg. Material studied in the MNHNL collection: East Gutland: Wilferdange, Conzefenn (1 ♂, 22.IX - 19.X.2000, Malaise); Minette: Kockelscheuer, Conter Jans Boesch (1 ♀, 22.VII - 5.VIII.1999; 2 ♀♀, 2.IX - 27.IX.1999, Malaise).

New record for Luxembourg.

Geographical distribution. Known till now only from a few countries in central and northern Europe (Austria, Belarus, Czech Republic, Germany, Great Britain, Sweden, Switzerland, The Netherlands) (Ossiannilsson 1992, Burckhardt 1994b, Burckhardt and Lauterer 1997a, Malenovský and Lauterer 2012, Serbina et al. 2015, den Bieman et al. 2019), but the species is probably more widespread on the European continent.

Biology. Reported till now only from *Salixelaeagnos* Scop. and *S.lapponum* L. (Salicaceae) (Ossiannilsson 1992, Burckhardt and Lauterer 1997a), but probably living also on other willows (*Salix* spp.) (Malenovský and Lauterer 2012, den Bieman et al. 2019). It spends the winter as an adult on shelter plants (conifers), on which it has been collected during many of its findings; probably performs only one generation per year.

***Eryngiofagalautereri* Loginova, 1977**

(Figs 4, 8, 16)

Findings in Luxembourg. Material studied in the MNHNL collection: East Gutland: Niederanven, Aarnescht (2 ♂♂, 27.V - 9.VI.1999, Malaise).

New record for Luxembourg.

Geographical distribution. Known till now only from a few countries in central-eastern Europe, *E.lautereri* has been described on material from the Czech Republic (Moravia) and Germany (Thuringia) (Loginova 1977), although the German material has been erroneously indicated in the original description as originating from Austria (according to Lauterer 1979). Later, it was reported from other localities in the Czech Republic and Germany, as well as from Slovakia (Lauterer 1979, Malenovský and Lauterer 2012). It seems to be a rare species.

Biology. Trophically linked to plants of the genus *Bupleurum* L. (Apiaceae) (Hodkinson, 2009), it is reported exclusively to live on *B.falcatum* L. (Loginova 1977, Lauterer 1979, Malenovský et al. 2011, Malenovský and Lauterer 2012). Life cycle of this psyllid has been studied by Lauterer (1979), who reports two generations per year, with adults emerging in May-June and in August-September, respectively (only one generation may be shown by part of the population, which spends summer as diapausing nymphs of the first offspring); winter is spent by the nymphs (of both generations) on the host plant.

***Lauritriozaalacris* (Flor, 1861)**

(Figs 4, 8, 12)

Findings in Luxembourg. No explicit mention exists in literature on findings of this insect in Luxembourg, though its galls produced on *Laurusnobilis* are reported by Lambinon and Schneider (2004) and by Schneider (2016) (West Gutland: Bonnevoie; Moselle: Remich), who did not report, however, any finding of this psyllid in or nearby the galls. Therefore, the present record is the first direct finding of *L.alacris* in Luxembourg.

Personal collection by the authors in: Oesling: West Gutland: Bertrange (1 ♀, 13 nymphs, 7.VI.2020, on *L.nobilis*).

Geographical distribution. Most probably native from the Mediterranean Region on wild laurel and widely distributed in this area (Halperin et al. 1982, Burckhardt 1988a, Burckhardt 1989, Conci et al. 1996, Zeidan-Gèze and Burckhardt 1998, Spodek et al. 2017), it has spread (and has been introduced) on cultivated *L.nobilis* in nurseries, gardens and parks throughout nearly all of Europe, including central and northern parts of the continent, up to Scandinavia (Hodkinson and White 1979, Hodkinson and White 1981, Burckhardt 1988c, Ossiannilsson 1992, Conci et al. 1996, O'Connor et al. 1997, Hollis and Martin 1997, Burckhardt and Mühlethaler 2003, Burckhardt 2005, Seljak 2006, Cocquempot 2008, O’Connor and Malumphy 2011, den Bieman et al. 2019), as well as eastwards to Anatolia (Turkey), the Caucasian Region, the Crimean Peninsula (Burckhardt 1989, Ossiannilsson 1992, Zeidan-Gèze and Burckhardt 1998, Burckhardt and Mühlethaler 2003) and westwards to North (USA) and South America (Argentina, Brazil, Chile) (Hodkinson and White 1981, Burckhardt 1988c, Hodkinson 1988, Mifsud et al. 2010, Burckhardt and Queiroz 2012, Percy et al. 2012).

Biology. According to the large body of literature on this species (Hodkinson and White 1979, Hodkinson and White 1981, Burckhardt 1983, Burckhardt 1988a, Burckhardt 1988b, Hodkinson 1988, Burckhardt 1989, Ossiannilsson 1992, Conci et al. 1996, Hollis and Martin 1997, O'Connor et al. 1997, Zeidan-Gèze and Burckhardt 1998, Burckhardt and Mühlethaler 2003, Burckhardt 2005, Seljak 2006, Cocquempot 2008, Hodkinson 2009, Marchini et al. 2012, Percy et al. 2012, Burckhardt and Queiroz 2012, Spodek et al. 2017), *L.alacris* is oligophagous on plants of the genus *Laurus* L. (Lauraceae), with *L.nobilis* L. being its most frequent host plant, but likely also living on other congeneric plant species. During spring and summer, adults are extremely active, flying intensively and spreading around from their host plants; this is why they can be found (and have been recorded in the past literature) on a great number of occasional plants. *Lauritriozaalacris* performs 1-2 generations per year, overwintering as an adult on the host plants. It causes leaf galls, by rolling their margins down to the lower surface (Houard 1908, Houard 1909, Houard 1913, Ross and Hedicke 1927, Buhr 1964, Buhr 1965, Sampò 1977).

Economic significance. In case of a strong infestation, leaf deformations and galls caused by this psyllid, which dry up and become black as soon as they are abandoned by the nymphs, may cause serious aesthetic damage to cultivated laurel in gardens and parks and direct control of this insect may be occasionally necessary, especially in nurseries and on young plants.

***Trichochermeswalkeri* (Foerster, 1848)**

Findings in Luxembourg. *Trichochermeswalkeri* was reported from Luxembourg by Baugnée (2001). In addition, the galls it causes on *Rhamnuscathartica* L. were reported for the country by Lambinon and Schneider (2004) (Moselle: Ahn, Wormeldange; Minette: Dudelange, Reckange-sur-Mess, Rumelange; West Gutland: Hamm, Steinfort, East Gutland: Rosport).

Geographical distribution. European chorotype, with a distribution almost limited to the central and northern part of the continent (Hodkinson and White 1979, Ossiannilsson 1992, Conci et al. 1996, Seljak 2006, Ripka 2008, Ripka 2009, Malenovský et al. 2011, Ouvrard et al. 2015, Serbina et al. 2015, Drohojowska and Klasa 2019, den Bieman et al. 2019).

Biology. Strictly oligophagous on *Rhamnus* spp. (Rhamnaceae), with records especially on *R.cathartica* L. (Hodkinson and White 1979, Burckhardt 1983, Ossiannilsson 1992, Conci et al. 1996, Seljak 2006, Ripka 2008, Ripka 2009, Malenovský et al. 2011), but also frequently recorded on *R.saxatilis* Jacq. (Cobau 1912, Ripka 2008). It performs one generation per year and overwinters as an egg on the host plants.

***Triozaabdominalis* Flor, 1861**

(Figs 4, 8, 12, 16)

Findings in Luxembourg. Material studied in the MNHNL collection: East Gutland: Wilferdange, Conzefenn (2 ♂♂, 4 ♀♀, 22.IX - 19.X.2000, Malaise).

New record for Luxembourg.

Geographical distribution. Widely distributed in Europe, especially in the north and central part of the continent (Hodkinson and White 1979, Burckhardt 1983, Ossiannilsson 1992, Conci et al. 1996, Seljak 2006, Gertsson 2010, Malenovský and Lauterer 2012, Malenovský et al. 2014, Ouvrard et al. 2015, Serbina et al. 2015, Drohojowska and Klasa 2019). Reports from central and east Asia, such as from the Korean Peninsula or Mongolia (Kwon 1983) need to be confirmed.

Biology. Based on data from the literature, *T.abdominalis* is widely oligophagous on plants belonging to the genera *Achillea* L., *Anthemis* L. and *Chrysanthemum* L. (Asteraceae) (Hodkinson and White 1979, Burckhardt 1983, Ossiannilsson 1992, Conci et al. 1996, Seljak 2006, Hodkinson 2009, Gertsson 2010, Malenovský and Lauterer 2012, Malenovský et al. 2014), with *Achilleamillefolium* L. being the most common host plant. *Triozaabdominalis* performs one generation per year and spends winter as an adult on shelter plants (conifers).

***Triozacentranthi* (Vallot, 1829)**

Findings in Luxembourg. No explicit mention exists in literature till now on findings of this insect in Luxembourg, though its galls produced on *Centranthusruber* are reported by Lambinon et al. (2012) (West Gutland: Bonnevoie, Buschdorf, Kirchberg; Minette: Dudelange, Tétange; Moselle: Mertert) and by Schneider (2016) (Oesling: Stolzembourg; West Gutland: Howald, Luxembourg; East Gutland: Schoos), who did not report, however, any finding of this psyllid in or nearby the galls. Therefore, the present record is the first direct finding of *T.centranthi* in Luxembourg.

Personal collection by the authors in: West Gutland, Strassen (1 ♀, 27.IV.2020, from general sweeping with net); Moselle: Remerschen (1 leaf gall with 3 nymphs inside, 28.V.2020, on *Centranthusruber*).

Geographical distribution. Euro-Mediterranean chorotype, distributed in central and eastern Europe (northwards up to Denmark and Great Britain; Hodkinson and White 1979, Burckhardt 1988a, Burckhardt 1989, Ossiannilsson 1992, Conci et al. 1996, Seljak 2006, Ripka 2008, Cocquempot 2008, Ouvrard et al. 2015, Spooner 2016, Badmin 2017, Drohojowska and Klasa 2019, den Bieman et al. 2019) and the Mediterranean Basin (Algeria, Morocco, Israel; Halperin et al. 1982, Burckhardt 1989, Spodek et al. 2017); distributed eastwards to Turkey and the Caucasian Region (Burckhardt 1989, Ossiannilsson 1992, Burckhardt and Önuçar 1993).

Biology. Widely oligophagous on several species of the genera *Centranthus* DC. [*C.angustifolius* (Mill.) DC., *C.calcitrapae* (L.) Dufr., *C.ruber* (L.) DC.], *Fedia* Gaertn. (*F.cornucopiae* Gaertn.) and *Valerianella* Mill. [*V.carinata* Loisel., *V.coronata* (L.) DC., *V.dentata* (L.) Pollich, *V.locusta* (L.) Laterr. (= *V.olitoria* (L.) Pollich), *V.rimosa* Bast.] (Caprifoliaceae) (Hodkinson and White 1979, Burckhardt 1988a, Burckhardt 1989, Ossiannilsson 1992, Conci et al. 1996, Seljak 2006, Cocquempot 2008, Ripka 2008, Hodkinson 2009, Spooner 2016, Badmin 2017). It usually performs one generation per year, overwintering as an adult on shelter plants (conifers), though in warmer areas, it likely shows an almost continuous life-cycle (with an undefined number of yearly generations), spending the winter in all developmental stages.

On its host plants, *T.centranthi* causes showy leaf deformations, by rolling up the margins and forming irregular and turgid galls, which are initially pale green and later turn red; flowers and inflorescences may be also deformed by this psyllid, through hypertrophies or atrophies of the stamens or distortion of apical flowers (which are reduced to subglobular processes) (Houard 1908, Houard 1909, Houard 1913, Sampò 1975, Conci et al. 1996).

***Triozacirsii* Löw, 1881**

(Figs 4, 8, 12, 16)

Findings in Luxembourg. Material studied in the MNHNL collection: East Gutland: Koedange, Poenn (8 ♂♂, 13 ♀♀, 8.VII - 22.VII.1999; 5 ♂♂, 12 ♀♀, 22.VII - 5.VIII.1999; 1 ♀, 19.VIII - 2.IX.1999; 8 ♂♂, 6 ♀♀, 2.IX - 27.IX.1999, Malaise).

New record for Luxembourg.

Geographical distribution. With the exception of the Balkan Peninsula (Bulgaria, Greece, Romania), findings of *T.cirsii* concern exclusively central-northern Europe (Burckhardt 1988a, Ossiannilsson 1992, Seljak 2006, Gertsson 2010, Malenovský et al. 2011, Ouvrard et al. 2015, Holzinger et al. 2017, Drohojowska and Klasa 2019).

Biology. *Triozacirsii* is oligophagous on Asteraceae of the genus *Cirsium* Mill., with records confirmed from *C.arvense* (L.) Scop., *C.erisithales* Scop., *C.heterophyllum* (L.) Hill, *C.oleraceum* Scop., *C.palustre* (L.) Scop. (Burckhardt 1988a, Ossiannilsson 1992, Seljak 2006, Hodkinson 2009, Malenovský et al. 2011). The number of yearly generations is unknown. It overwinters as adult on shelter plants (conifers).

***Triozaflavipennis* Foerster, 1848**

Findings in Luxembourg. This species has not been found by the authors in Luxembourg so far. Nevertheless, findings of its leaf deformations produced on *Aegopodiumpodagraria* are reported by Lambinon and Schneider (2004) and by Burton et al. (2019) (West Gutland: Hollenfels, East Gutland: Manternach), who also mentioned the occurrence of eggs and nymphs of this psyllid species in the above galls, thus explicitly recording its occurrence in Luxembourg.

Geographical distribution. *Triozaflavipennis* is a typical European chorotype, being distributed mainly in the central and northern areas of the continent (Hodkinson and White 1979, Ossiannilsson 1992, Conci et al. 1996, Hansen and Greve 1999, Seljak 2006, Ripka 2008, Malumphy et al. 2009, Gertsson 2010, Ripka 2012, Serbina et al. 2015, Drohojowska and Klasa 2019, den Bieman et al. 2019).

Biology. Monophagous on *Aegopodiumpodagraria* L. (Apiaceae) (Hodkinson and White 1979, Ossiannilsson 1992, Conci et al. 1996, Hansen and Greve 1999, Seljak 2006, Ripka 2008, Malumphy et al. 2009). It performs likely only one generation per year and overwinters as an adult on shelter plants (conifers). On leaves of its host plant, this psyllid produces small pit-galls, protruding to the upper face (Houard 1908, Houard 1909, Cobau 1912, Houard 1913, Ross and Hedicke 1927, Buhr 1964, Buhr 1965, Lauterer 1991).

***Triozagalii* Foerster, 1848**

(Figs 4, 8, 12, 16)

Findings in Luxembourg. Personal field collection by the authors: Moselle: Bech/Kleinmacher (1 ♀, 30.VII.2019, on *Fraxinusexcelsior*, occasional plant; 3 ♀♀, 8.V.2020, from general sweeping with net).

Material studied in the MNHNL collection: East Gutland: Koedange, Poenn (1 ♂, 8.VII - 22.VII.1999, Malaise); Moselle: Canach, Wéngertsbierg (1 ♂, 25.VI - 8.VII.1999, Malaise).

New record for Luxembourg.

Geographical distribution. A large number of literature records is available for this species for nearly the entire Palaearctic Region; nevertheless, due to a recent assessment of the *T.galii* species group (Burckhardt and Lauterer 2006), many reports from the East Palaearctic Region may refer to other taxa. Therefore, based on the presently reliable information, a Western Palaearctic distribution extending to central Asia should be referred to for this psyllid.

Biology. Widely oligophagous on numerous Rubiaceae of the genera *Asperula* L., *Cruciata* Mill., *Galium* L., *Rubia* L. and *Sherardia* L. (Hodkinson and White 1979, Hodkinson and Hollis 1981, Hodkinson 1983, Burckhardt 1988a, Burckhardt 1989, Ossiannilsson 1992, Conci et al. 1996, Burckhardt 2005, Burckhardt and Lauterer 2006, Seljak 2006, Ripka 2008, Hodkinson 2009, Malenovský et al. 2011, Newbould 2012, Spodek et al. 2017, den Bieman et al. 2019). Literature offers numerous different descriptions of its life-cycle, probably deriving from the taxonomic confusion that has only recently been unravelled; based on the reliable information available today, *T.galii* very likely overwinters as an adult, but it is not possible to indicate details on its biology, including the number of yearly generations (Burckhardt and Lauterer 2006). Similarly, the gall-forming activity of this species being confirmed, the considerable quantity of cecidia described and attributed to *T.galii* (deformation and redness of terminal leaves, shortening of internodes, rolling up of the leaf margins, hypogean cecidia on rhizomes etc.) (Houard 1908, Houard 1909, Houard 1913, Ross and Hedicke 1927, Docters van Leeuwen 1937, Buhr 1964, Buhr 1965) is most likely to be referred to different species of the group.

***Triozaremota* Foerster, 1848**

(Figs 4, 8, 12, 16)

Findings in Luxembourg. Galls produced by this psyllid on *Quercusrobur* L. are reported by Lambinon and Schneider (2004) and by Schneider (2016) (Oesling: Troisvierges; West Gutland: Bonnevoie, Biergerkräiz, Howald, Kirchberg, Luxembourg, Steinfort; East Gutland: Oberanven; Minette: between Bergem and Schifflange, Dudelange, Esch-sur-Alzette, Frisange, Kayl, Schifflange; Moselle: between Gare d’Ellange and Altwies, Mondorf-les-Bains). The latter author also refers to the occurrence of an insect nymph within each pit gall, thus explicitly mentioning the finding of this insect in Luxembourg.

Personal collection by the authors in: Oesling: Arsdorf (21 nymphs, 22.VIII.2019, on *Quercusrobur*); West Gutland: Strassen (2 pit-galls with nymphs, 16.VIII.2020, on *Quercus* sp.).

Material studied in the MNHNL collection: Oesling: Basbellain, Klengelbaach (3 ♂♂, 7 ♀♀, 22.IX - 19.X.2000, Malaise), Eselborn, Bréichen (2 ♂♂, 2 ♀♀, 19.X - 7.XI.2000, Malaise), Goebelsmühle (5 ♂♂, 11 ♀♀, 22.IX - 19.X.2000, Malaise), Hoscheid, Molberlay (2 ♂♂, 1 ♀, 22.IX - 19.X.2000, Malaise), Lellingen, Op Baerel (2 ♂♂, 2 ♀♀, 22.IX - 19.X.2000, Malaise); West Gutland: Capellen, Werwelslach (1 ♀, 13.IV - 22.IV.1999, Malaise); East Gutland: Godbrange, Schléidelbierg (1 ♂, 1 ♀, 6.IV - 22.IV.1999; 1 ♀, 22.IV - 11.V.1999, Malaise), Wilferdange, Conzefenn (2 ♀♀, 22.IX - 19.X.2000, Malaise); Minette: Kockelscheuer, Conter Jans Boesch (1 ♀, 13.IV - 22.IV.1999; 1 ♂, 2.IX - 27.IX.1999, Malaise), Schifflange, Kayl, Brucherbierg (1 ♀, 22.IV - 11.V.1999, Malaise).

Geographical distribution. *Triozaremota* shows a West Palaearctic distribution, with records from most parts of Europe and the Mediterranean Region (Hodkinson and White 1979, Burckhardt 1989, Ossiannilsson 1992, Conci et al. 1996, Seljak 2006, Ripka 2008, Malumphy et al. 2009, Gertsson 2010, Malenovský et al. 2011, O’Connor and Malumphy 2011, Serbina et al. 2015, Spodek et al. 2017, Drohojowska and Klasa 2019, den Bieman et al. 2019), eastwards to Anatolia, the Caucasian Region and Iran (Burckhardt 1989, Önuçar and Ulu 1991, Ossiannilsson 1992, Burckhardt and Lauterer 1993, Burckhardt and Önuçar 1993, Malumphy et al. 2009). Records from Japan (Burckhardt 1989, Önuçar and Ulu 1991, Ossiannilsson 1992, Burckhardt and Lauterer 1993, Burckhardt and Önuçar 1993, Malumphy et al. 2009) would require further verification.

Biology. Strictly oligophagous on many deciduous species of the genus *Quercus* L. (Fagaceae), with most frequent records on *Q.petraea* (Matt.) Liebl. and *Q.robur* L. (Hodkinson and White 1979, Burckhardt 1988a, Burckhardt 1989, Önuçar and Ulu 1991, Ossiannilsson 1992, Conci et al. 1996, Zeidan-Gèze and Burckhardt 1998, Seljak 2006, Ripka 2008, Hodkinson 2009, Malumphy et al. 2009, Malenovský et al. 2011, Spodek et al. 2017). According to Lauterer (1991), it performs only one generation per year, overwintering as an adult on shelter plants (conifers) and with a very slow nymphal development during summer (from May to late August), which is spent as a 2^nd^ instar nymph in a sort of “parapause”; aestivating nymphs soon resume their development in September and produce adults already in October (the latter overwinter).

This psyllid produces little pit galls on the leaves of its host plants, protruding to the upper face; the nymph causing each gall settles on the corresponding concavity occurring on the lower face (Houard 1908, Houard 1909, Houard 1913, Ross and Hedicke 1927, Buhr 1964, Buhr 1965).

***Triozarhamni* (Schrank, 1801)**

Findings in Luxembourg. No explicit mention exists in literature on findings of this insect in Luxembourg, though its galls produced on *Rhamnuscathartica* L. are reported by Lambinon and Schneider (2004) (Moselle: Ahn), who did not report, however, any finding of this psyllid in or nearby the galls.

This species has not been found by us during our research in 2019-2020 nor in the investigated Malaise and Moericke trap samples; therefore, based on finding of its galls, it likely occurs in Luxembourg, but its presence needs to be confirmed by direct observations.

Geographical distribution. *Triozarhamni* is a European chorotype, distributed and recorded over nearly the whole continent eastwards to Turkey and the Caucasian Region (Hodkinson and White 1979, Burckhardt 1988b, Ossiannilsson 1992, Burckhardt and Önuçar 1993, Conci et al. 1996, Hansen and Greve 1999, Seljak 2006, Ripka 2008, Ripka 2009, Gertsson 2010, Malenovský et al. 2011, Kanturski and Drohojowska 2013, Ouvrard et al. 2015).

Biology. Strictly oligophagous on *Rhamnus* spp., with *R.cathartica* L. being its most frequent host plant, but also reported on *Rhamnuspallasii* Fisch. & C.A. Mey. (Hodkinson and White 1979, Burckhardt 1983, Burckhardt 1988b, Ossiannilsson 1992, Conci et al. 1996, Hansen and Greve 1999, Seljak 2006, Ripka 2008, Ripka 2009, Malenovský et al. 2011, Kanturski and Drohojowska 2013). It probably performs two generations per year (Lauterer 1991) and overwinters as an adult on shelter plants (conifers). As a result of egg deposition, *T.rhamni* causes small pit-galls on its host plant leaves (Houard 1908, Houard 1909, Cobau 1912, Houard 1913, Ross and Hedicke 1927, Buhr 1964, Buhr 1965).

***Triozaurticae* (Linnaeus, 1758)**

(Figs 4, 8, 12, 16)

Findings in Luxembourg. Galls produced by this species on *Urticadioica* are reported by Lambinon et al. (2012) (Oesling: between Perlé and Holtz, Troisvierges; Minette: Dudelange; Moselle: Remich; West Gutland: Bonnevoie, Gantenbeinsmühle, Schleifmühle, Steinfort; East Gutland: Bleesbréck, Gare de Sandweiler-Contern), who did not report, however, any finding of this psyllid in or nearby the galls. Therefore, the present record is the first direct finding of *T.urticae* in Luxembourg.

Personal collection by the authors in: Oesling: West Gutland: Arsdorf (1♀, 20.V.2020, from general sweeping with net; 1 ♂, 1 ♀, 8.VI.2020, on *Urticadioica* L.), Brouch (1♀, 6.IX.2019, from general sweeping with net); Minette: Kayl/Tetange (13 ♂♂, 16 ♀♀, 23.VII.2019, on *U.dioica*).

Material studied in the MNHNL collection: Oesling: Eselborn, Bréichen (1 ♂, 19.X - 7.XI.2000, Malaise); West Gutland: Capellen, Engelsratt (4 ♂♂, 3 ♀♀, 9.VI - 25.VI.1999; 15 ♂♂, 10 ♀♀, 25.VI - 8.VII.1999; 6 ♂♂, 8 ♀♀, 8.VII - 22.VII.1999; 5 ♂♂, 4 ♀♀, 22.VII - 5.VIII.1999, Malaise); East Gutland: Niederanven, Aarnescht (1 ♀, 9.VI - 25.VI.1999, Malaise), Waldhausenerdickt, Sauerwisen (5 ♂♂, 15 ♀♀, 22.IX - 19.X.2000, Malaise), Wilferdange, Conzefenn (1 ♀, 3.VIII - 24.VIII.2000, Malaise); Minette: Kockelscheuer, Conter Jans Boesch (1 ♂, 19.VIII - 2.IX.1999, Malaise); Moselle: Canach, Wéngertsbierg (1 ♂, 9.VI - 25.VI.1999, Malaise).

Geographical distribution. Widely distributed in the Palaearctic Region, from the Azores to Japan, being one of the psyllid species with the highest number of records reported in literature (amongst many others: Mathur 1975, Hodkinson and White 1979, Burckhardt 1988a, Burckhardt 1989, Halperin et al. 1988, Ossiannilsson 1992, Burckhardt and Lauterer 1993, Burckhardt and Önuçar 1993, Conci et al. 1996, Zeidan-Gèze and Burckhardt 1998, Aguiar and Martin 2001, Burckhardt 2005, Seljak 2006, Tishetshkin 2007, Labina 2008, Ripka 2008, Ripka and Kiss 2008, Malumphy et al. 2009, Gertsson 2010, Lauterer 2011, O’Connor and Malumphy 2011, Malenovský et al. 2011, Malenovský et al. 2012, Kanturski and Drohojowska 2013, Ouvrard et al. 2015, Serbina et al. 2015, Zendedel et al. 2016, Holzinger et al. 2017, Spodek et al. 2017, Haapalainen et al. 2018, Drohojowska and Klasa 2019, den Bieman et al. 2019

Biology. Strictly oligophagous on *Urtica* spp. (Urticaceae), with many records especially from *U.dioica* L. and *U.urens* L., but also from other plant species having a more restricted geographical distribution within the wide distribution area of this psyllid. *Triozaurticae* has a relatively rapid life cycle (depending on environmental conditions) and performs various generations per year (even more than four) on its host plants; it overwinters as an adult on various shelter plants, especially conifers.

This psyllid species may sometimes cause deformations to plants, by wrinkling or bubbling their leaves (Houard 1908, Houard 1909, Houard 1913, Ross and Hedicke 1927, Zangheri 1954, Buhr 1964, Buhr 1965, Sampò 1975).

## Final remarks

The research here presented, realised through field collections and the study of historical collection material stored at the MNHNL, allowed us to significantly increase the number of species of Psylloidea known for Luxembourg. Based on data available in literature so far and considering also the species already known in the country only by the records of their galls or deformations, the Luxembourg psyllid fauna has more than tripled as a result of the present work. The increase is even greater (almost 7-fold) if the number of species previously known for the territory is strictly limited to only those reported through the direct finding of specimens. As to methodological aspects, it is interesting to note that the contribution of passive collection - in particular Malaise trapping - added seven new species to our assessment.

Despite the new records here presented, our knowledge of the psyllid fauna of Luxembourg is still incomplete. Further sampling is required on potential host plants on which psyllids have not been collected so far. Thus, for example, research on plants of the genus *Sorbus* L. must be intensified, as well as on numerous herbaceous plants that host in Europe psyllid species of the genera *Craspedolepta* Enderlein, *Bactericera* Puton and *Trioza* Foerster; on willows (*Salix* spp.), further research could also allow us to find additional psyllid species. Considering the floristic richness occurring in Luxembourg and also the psyllid fauna of neighbouring or close countries which are better explored, despite its small size, it is likely that further research could lead to a significant increase of up to about 20% of the psyllid species found so far in Luxembourg.

Finally, on the applied level, all species living on agricultural crops deserve appropriate future attention, especially those belonging to species complexes recognised as vectors of phytopathogenic organisms and whose real distribution and harmfulness in Luxembourg should be suitably monitored. This can be realised by involving local plant protection services, thus to further investigate possible impacts of changing environments on biology, vectoring activity, pest importance and spread of individual species.

## Figures and Tables

**Figure 1.**
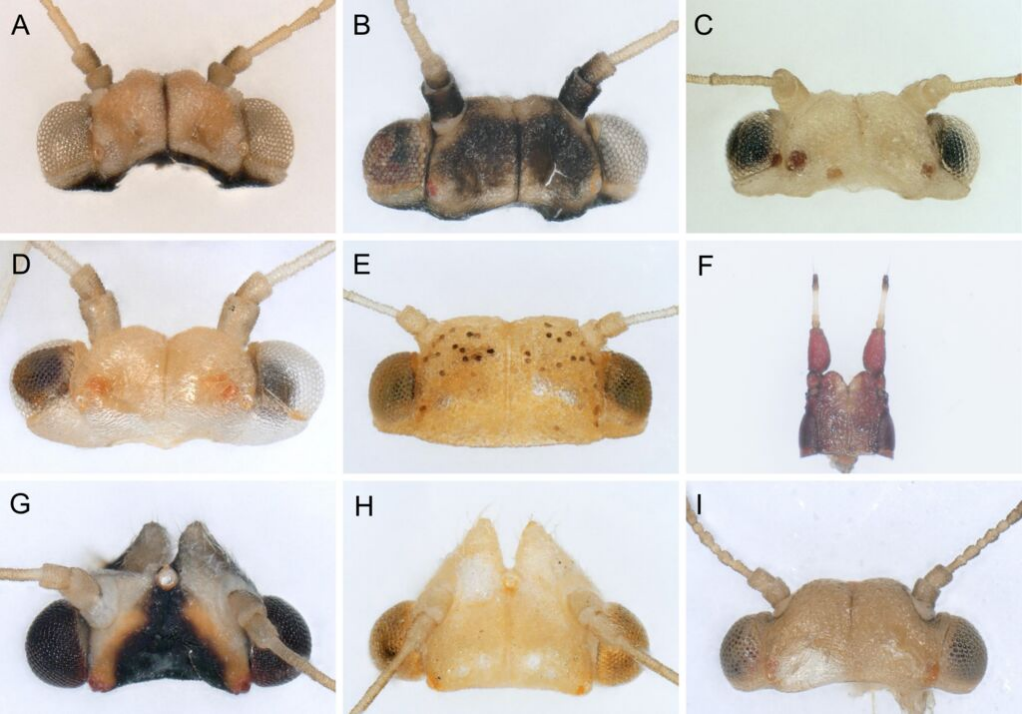
Overview of head structures of psyllid species in Luxembourg (Aphalaridae, Liviidae). **A** Aphalarasp.gr.polygoni Foerster, 1848 **B** *Craspedoleptanebulosa* (Zetterstedt, 1828) **C** *Craspedoleptanervosa* (Foerster, 1848) **D** *Craspedoleptasubpunctata* (Foerster, 1848) **E** *Rhinocolaaceris* (Linnaeus, 1758) **F** *Liviajunci* (Schrank, 1789) **G** *Psyllopsisfraxini* (Linnaeus, 1758) **H** *Psyllopsisfraxinicola* (Foerster, 1848) **I** *Strophingiaericae* (Curtis, 1835).

**Figure 2.**
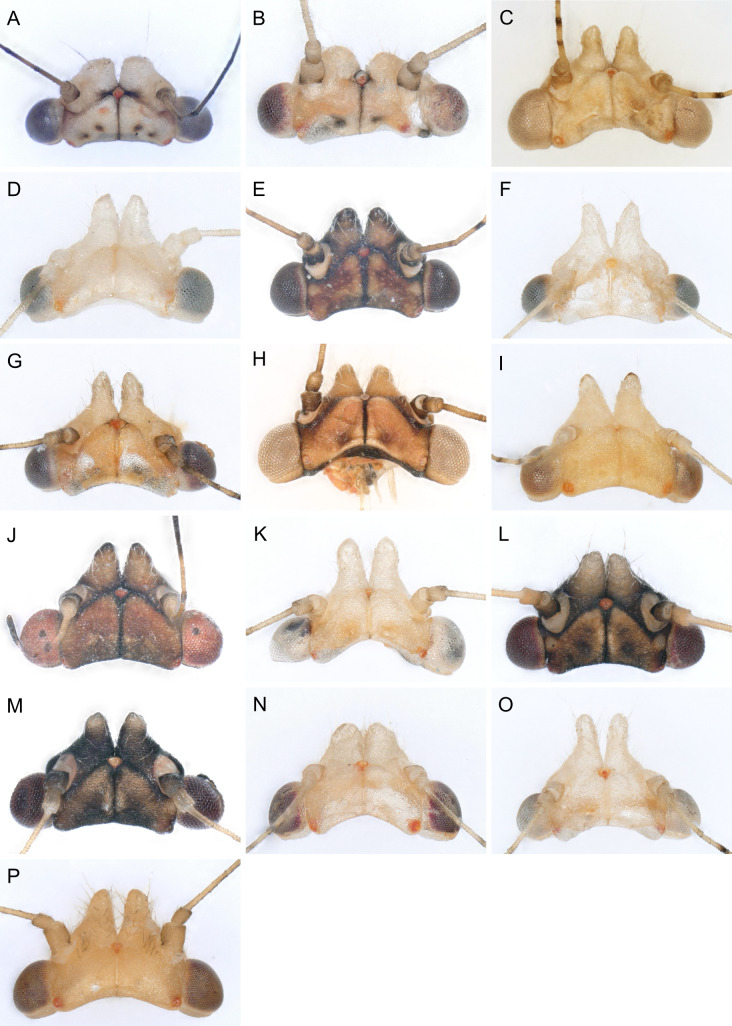
Overview of head structures of psyllid species in Luxembourg (Psyllidae I). **A** *Arytainagenistae* (Latreille, 1804) **B** *Arytainillaspartiophila* (Foerster, 1848) **C** *Cacopsyllaaffinis* (Löw, 1880) **D** *Cacopsyllaambigua* (Foerster, 1848) **E** *Cacopsyllacrataegi* (Schrank, 1801) **F** *Cacopsyllamali* (Schmidberger, 1836) **G** *Cacopsyllamelanoneura* (Foerster, 1848) **H** *Cacopsyllanigrita* (Zetterstedt, 1828) **I** *Cacopsyllaperegrina* (Foerster, 1848) **J** Cacopsyllasp.gr.pruni (Scopoli, 1763) **K** *Cacopsyllapulchra* (Zetterstedt, 1838) **L** *Cacopsyllapyri* (Linnaeus, 1758) **M** *Cacopsyllapyricola* (Foerster, 1848) **N** *Cacopsyllapyrisuga* (Foerster, 1848) **O** *Cacopsyllarhamnicola* (Scott, 1876) **P** *Cacopsyllavisci* (Curtis, 1835).

**Figure 3.**
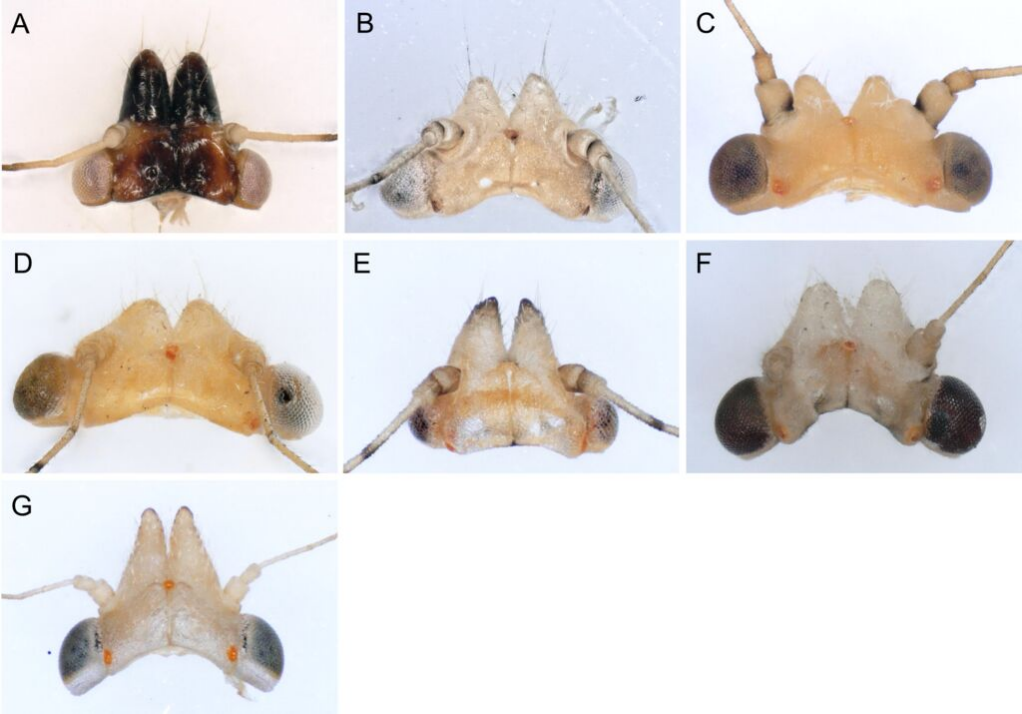
Overview of head structures of psyllid species in Luxembourg (Psyllidae II). **A** *Livillaulicis* Curtis, 1836 **B** *Psyllaalni* (Linnaeus, 1758) **C** *Psyllabetulae* (Linnaeus, 1758) **D** *Psyllafoersteri* Flor, 1861 **E** *Psyllahartigii* Flor, 1861 **F** *Spanioneurabuxi* (Linnaeus, 1758) **G** *Spanioneurafonscolombii* Foerster, 1848.

**Figure 4.**
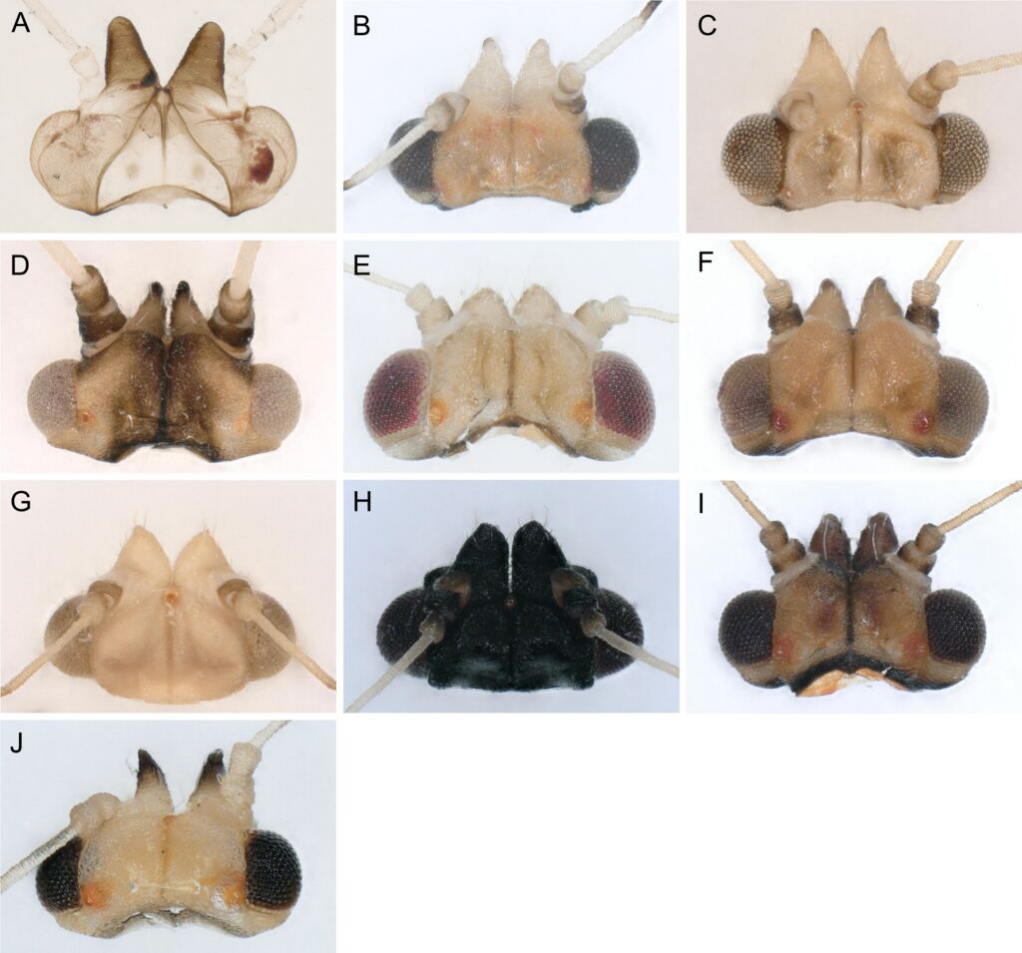
Overview of head structures of psyllid species in Luxembourg (Triozidae). **A** *Bactericeraalbiventris* (Foerster, 1848) **B** *Bactericeracurvatinervis* (Foerster, 1848) **C** *Bactericerasubstriola* Ossiannilsson, 1992 **D** *Eryngiofagalautereri* Loginova, 1977 **E** *Lauritriozaalacris* (Flor, 1861) **F** *Triozaabdominalis* Flor, 1861 **G** *Triozacirsii* Löw, 1881 **H** *Triozagalii* Foerster, 1848 **I** *Triozaremota* Foerster, 1848 **J** *Triozaurticae* (Linnaeus, 1758).

**Figure 5.**
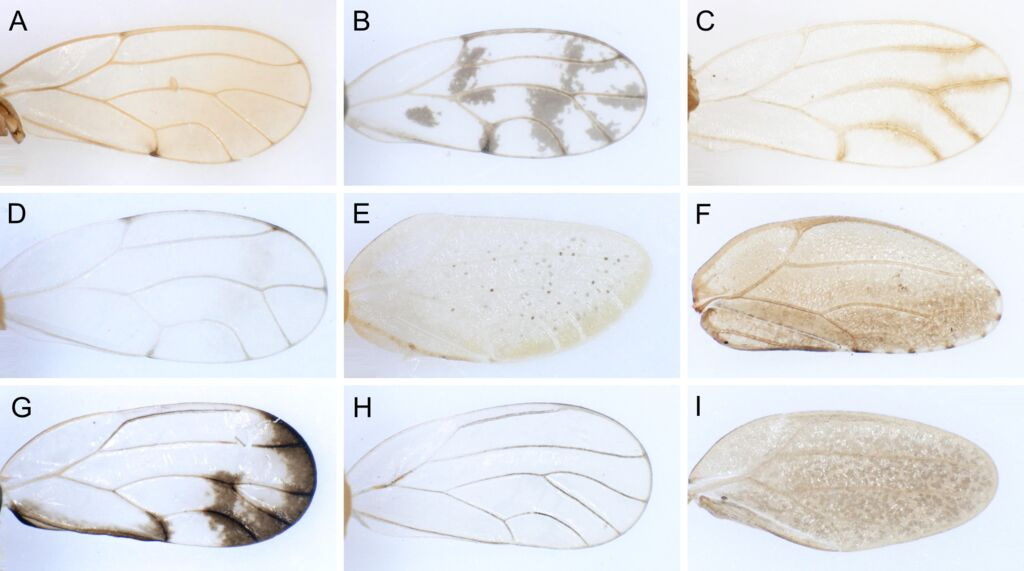
Overview of forewings of psyllid species in Luxembourg (Aphalaridae, Liviidae). **A** Aphalarasp.gr.polygoni Foerster, 1848 **B** *Craspedoleptanebulosa* (Zetterstedt, 1828) **C** *Craspedoleptanervosa* (Foerster, 1848) **D** *Craspedoleptasubpunctata* (Foerster, 1848) **E** *Rhinocolaaceris* (Linnaeus, 1758) **F** *Liviajunci* (Schrank, 1789) **G** *Psyllopsisfraxini* (Linnaeus, 1758) **H** *Psyllopsisfraxinicola* (Foerster, 1848) **I** *Strophingiaericae* (Curtis, 1835).

**Figure 6.**
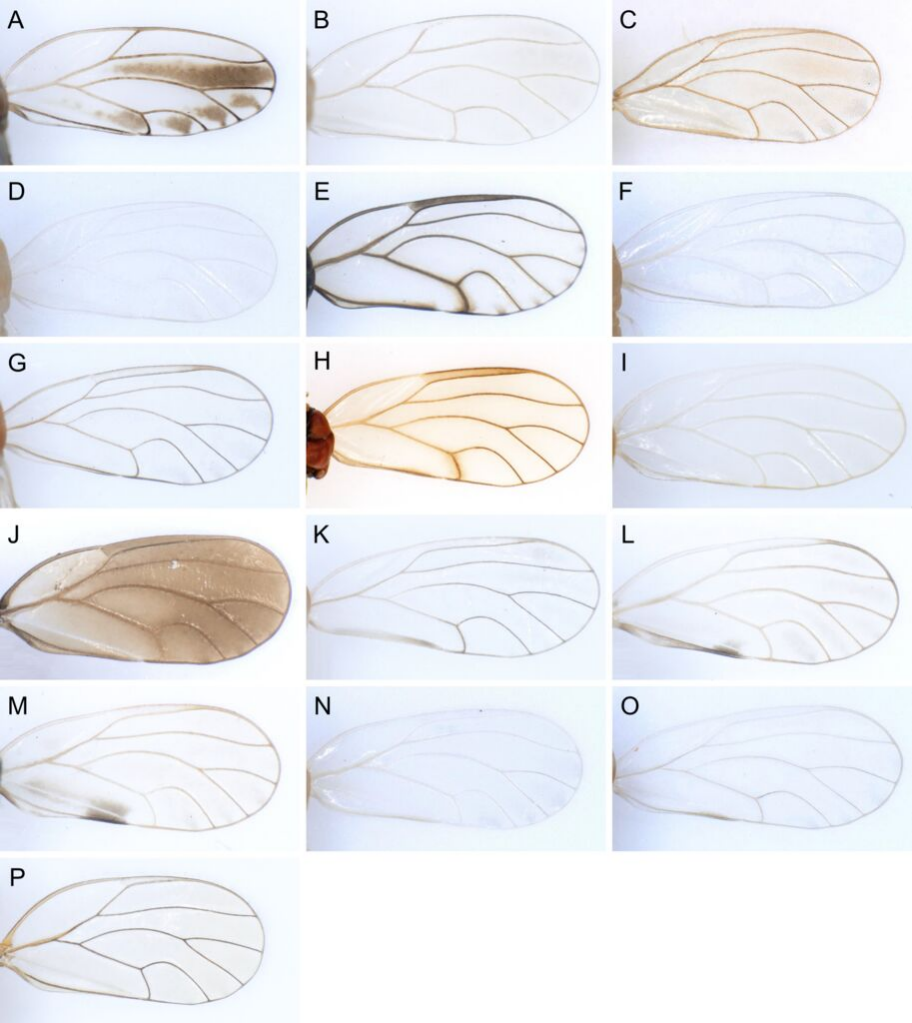
Overview of forewings of psyllid species in Luxembourg (Psyllidae I). **A** *Arytainagenistae* (Latreille, 1804) **B** *Arytainillaspartiophila* (Foerster, 1848) **C** *Cacopsyllaaffinis* (Löw, 1880) **D** *Cacopsyllaambigua* (Foerster, 1848) **E** *Cacopsyllacrataegi* (Schrank, 1801) **F** *Cacopsyllamali* (Schmidberger, 1836) **G** *Cacopsyllamelanoneura* (Foerster, 1848) **H** *Cacopsyllanigrita* (Zetterstedt, 1828) **I** *Cacopsyllaperegrina* (Foerster, 1848) **J** Cacopsyllasp.gr.pruni (Scopoli, 1763) **K** *Cacopsyllapulchra* (Zetterstedt, 1838) **L** *Cacopsyllapyri* (Linnaeus, 1758) **M** *Cacopsyllapyricola* (Foerster, 1848) **N** *Cacopsyllapyrisuga* (Foerster, 1848) **O** *Cacopsyllarhamnicola* (Scott, 1876) **P** *Cacopsyllavisci* (Curtis, 1835).

**Figure 7.**
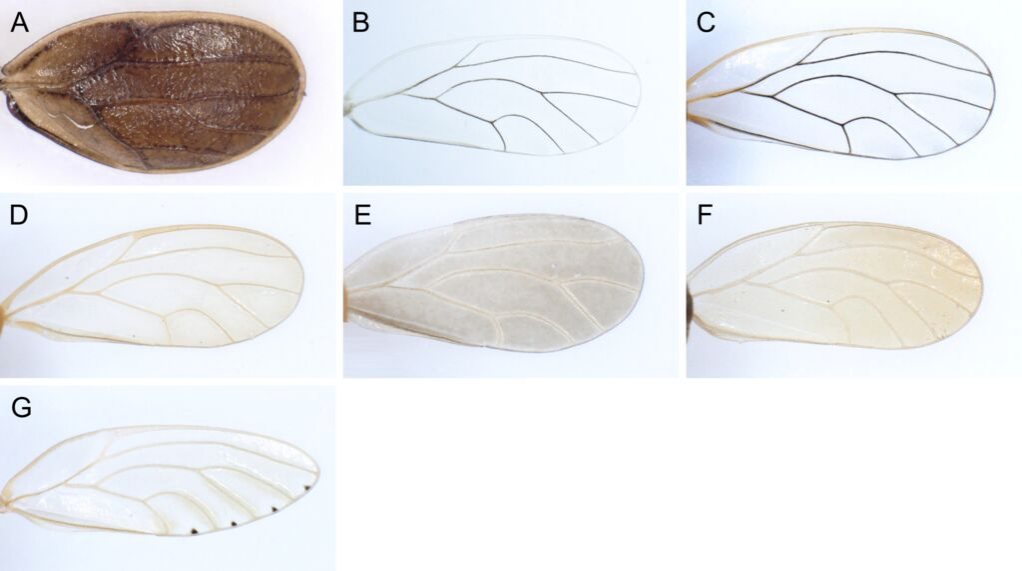
Overview of forewings of psyllid species in Luxembourg (Psyllidae II). **A** *Livillaulicis* Curtis, 1836 **B** *Psyllaalni* (Linnaeus, 1758) **C** *Psyllabetulae* (Linnaeus, 1758) **D** *Psyllafoersteri* Flor, 1861 **E** *Psyllahartigii* Flor, 1861 **F** *Spanioneurabuxi* (Linnaeus, 1758) **G** *Spanioneurafonscolombii* Foerster, 1848.

**Figure 8.**
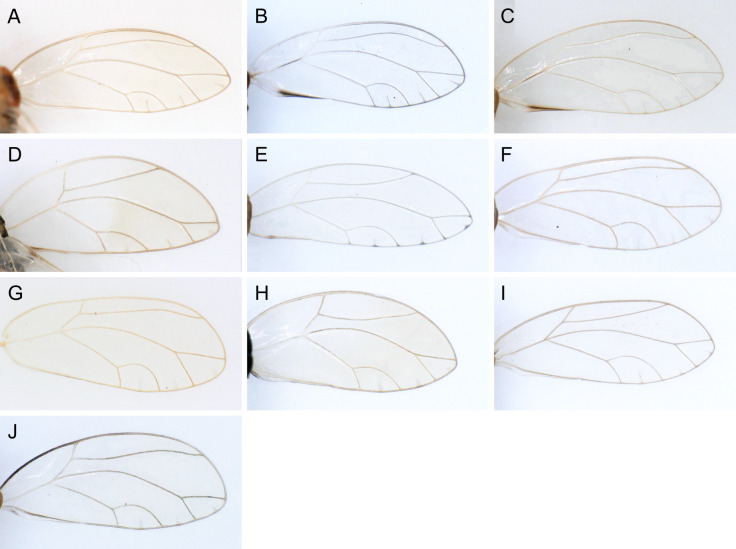
Overview of forewings of psyllid species in Luxembourg (Triozidae). **A** *Bactericeraalbiventris* (Foerster, 1848) **B** *Bactericeracurvatinervis* (Foerster, 1848) **C** *Bactericerasubstriola* Ossiannilsson, 1992 **D** *Eryngiofagalautereri* Loginova, 1977 **E** *Lauritriozaalacris* (Flor, 1861) **F** *Triozaabdominalis* Flor, 1861 **G** *Triozacirsii* Löw, 1881 **H** *Triozagalii* Foerster, 1848 **I** *Triozaremota* Foerster, 1848 **J** *Triozaurticae* (Linnaeus, 1758).

**Figure 9.**
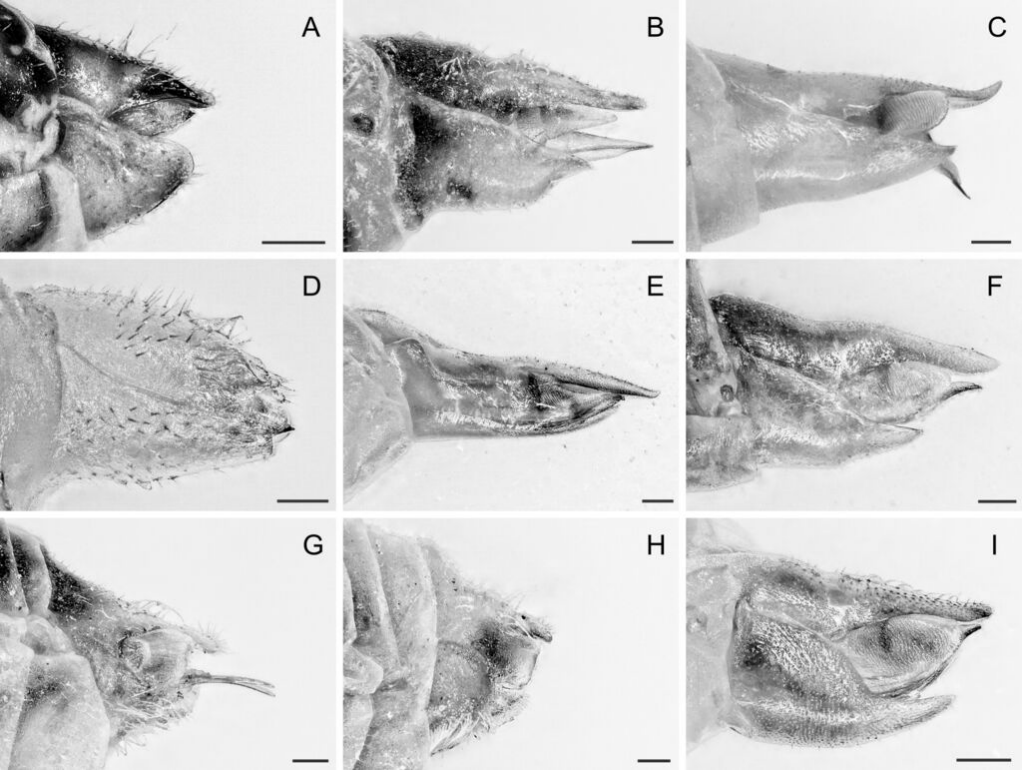
Overview of female terminalia of psyllid species in Luxembourg (Aphalaridae, Liviidae). **A** Aphalarasp.gr.polygoni Foerster, 1848 **B** *Craspedoleptanebulosa* (Zetterstedt, 1828) **C** *Craspedoleptanervosa* (Foerster, 1848) **D** *Craspedoleptasubpunctata* (Foerster, 1848) **E** *Rhinocolaaceris* (Linné, 1758) **F** *Liviajunci* (Schrank, 1789) **G** *Psyllopsisfraxini* (Linnaeus, 1758) **H** *Psyllopsisfraxinicola* (Foerster, 1848) **I** *Strophingiaericae* (Curtis, 1835).

**Figure 10.**
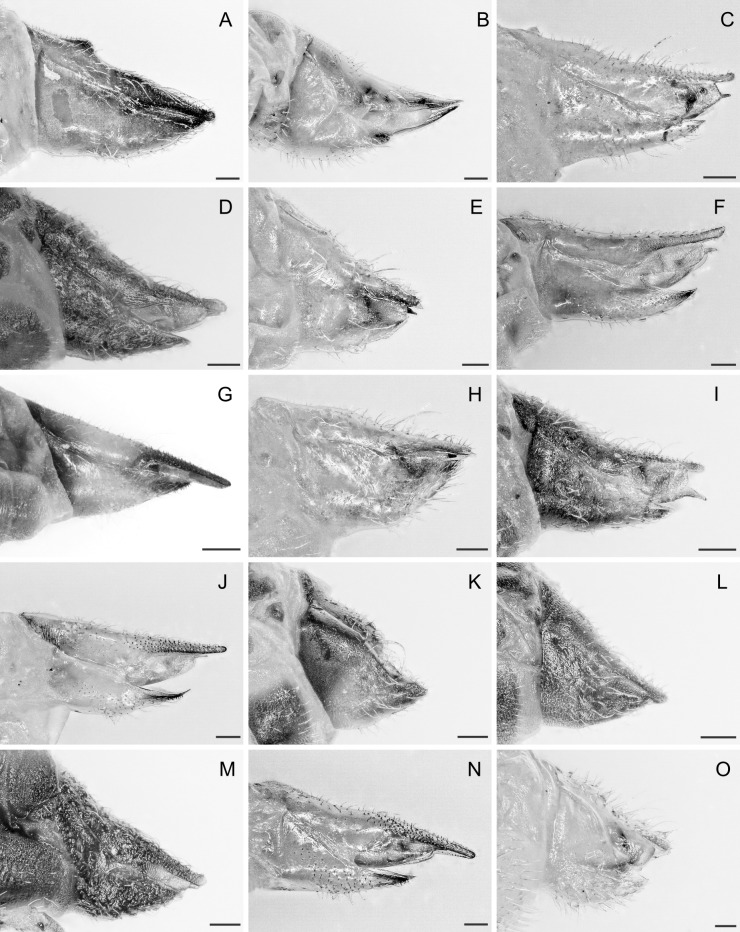
Overview of female terminalia of psyllid species in Luxembourg (Psyllidae I). **A** *Arytainagenistae* (Latreille, 1804) **B** *Arytainillaspartiophila* (Foerster, 1848) **C** *Cacopsyllaambigua* (Foerster, 1848) **D** *Cacopsyllacrataegi* (Schrank, 1801) **E** *Cacopsyllamali* (Schmidberger, 1836) **F** *Cacopsyllamelanoneura* (Foerster, 1848) **G** *Cacopsyllanigrita* (Zetterstedt, 1828) **H** *Cacopsyllaperegrina* (Foerster, 1848) **I** Cacopsyllasp.gr.pruni (Scopoli, 1763) **J** *Cacopsyllapulchra* (Zetterstedt, 1838) **K** *Cacopsyllapyri* (Linnaeus, 1758) **L** *Cacopsyllapyricola* (Foerster, 1848) **M** *Cacopsyllapyrisuga* (Foerster, 1848) **N** *Cacopsyllarhamnicola* (Scott, 1876) **O** *Cacopsyllavisci* (Curtis, 1835).

**Figure 11.**
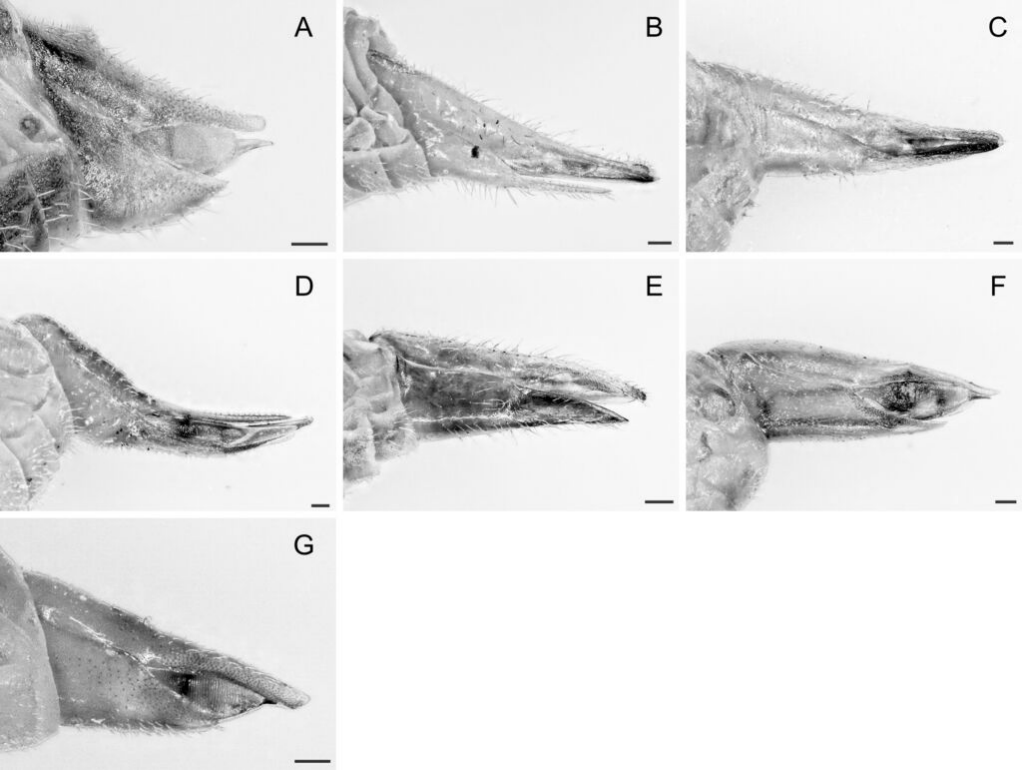
Overview of female terminalia of psyllid species in Luxembourg (Psyllidae II). **A** *Livillaulicis* Curtis, 1836 **B** *Psyllaalni* (Linnaeus, 1758) **C** *Psyllabetulae* (Linnaeus, 1758) **D** *Psyllafoersteri* Flor, 1861 **E** *Psyllahartigii* Flor, 1861 **F** *Spanioneurabuxi* (Linnaeus, 1758) **G** *Spanioneurafonscolombii* Foerster, 1848.

**Figure 12.**
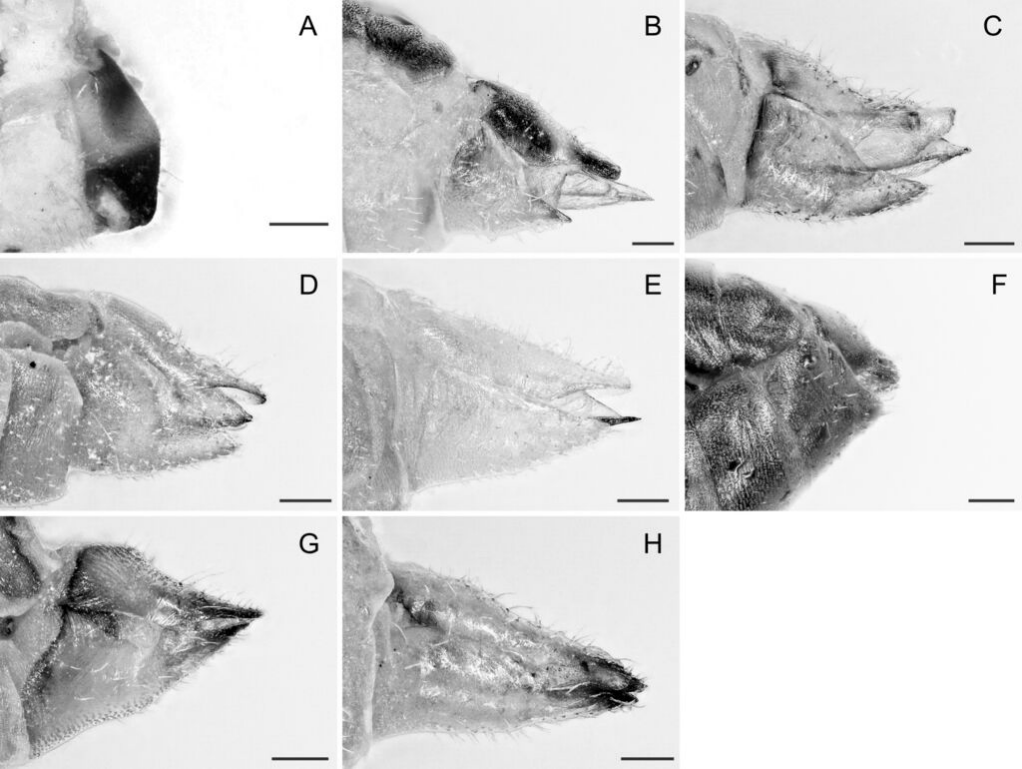
Overview of female terminalia of psyllid species in Luxembourg (Triozidae). **A** *Bactericeraalbiventris* (Foerster, 1848) **B** *Bactericeracurvatinervis* (Foerster, 1848) **C** *Lauritriozaalacris* (Flor, 1861) **D** *Triozaabdominalis* Flor, 1861 **E** *Triozacirsii* Löw, 1881 **F** *Triozagalii* Foerster, 1848 **G** *Triozaremota* Foerster, 1848 **H** *Triozaurticae* (Linnaeus, 1758).

**Figure 13.**
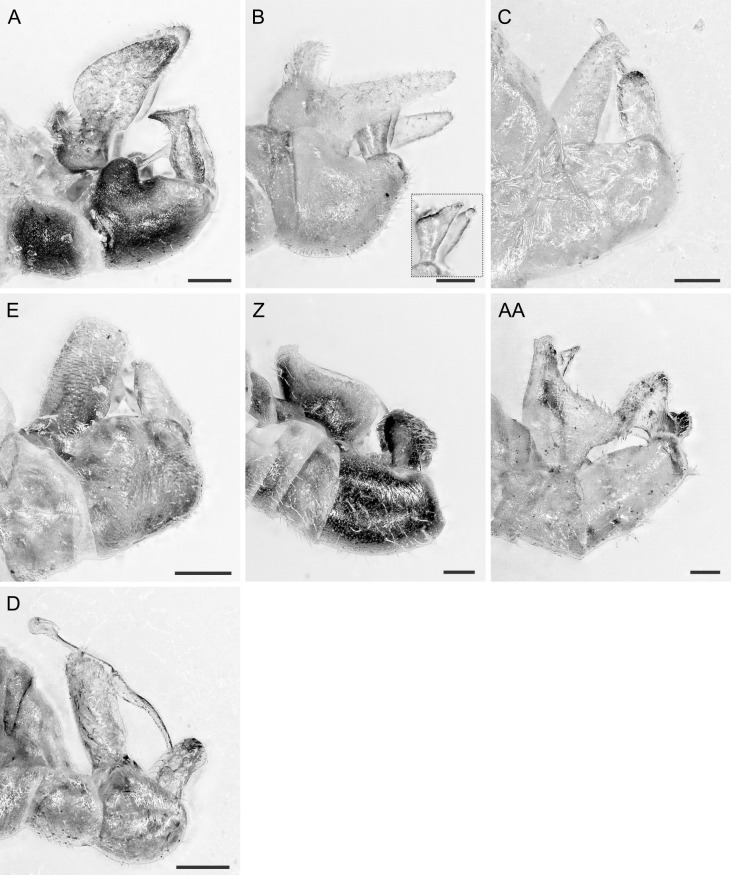
Overview of male terminalia of psyllid species in Luxembourg (Aphalaridae, Liviidae). **A** *Craspedoleptanebulosa* (Zetterstedt, 1828) **B** *Craspedoleptasubpunctata* (Foerster, 1848) **C** *Rhinocolaaceris* (Linnaeus, 1758) **D** *Liviajunci* (Schrank, 1789) **E** *Psyllopsisfraxini* (Linnaeus, 1758) **F** *Psyllopsisfraxinicola* (Foerster, 1848) **G** *Strophingiaericae* (Curtis, 1835).

**Figure 14.**
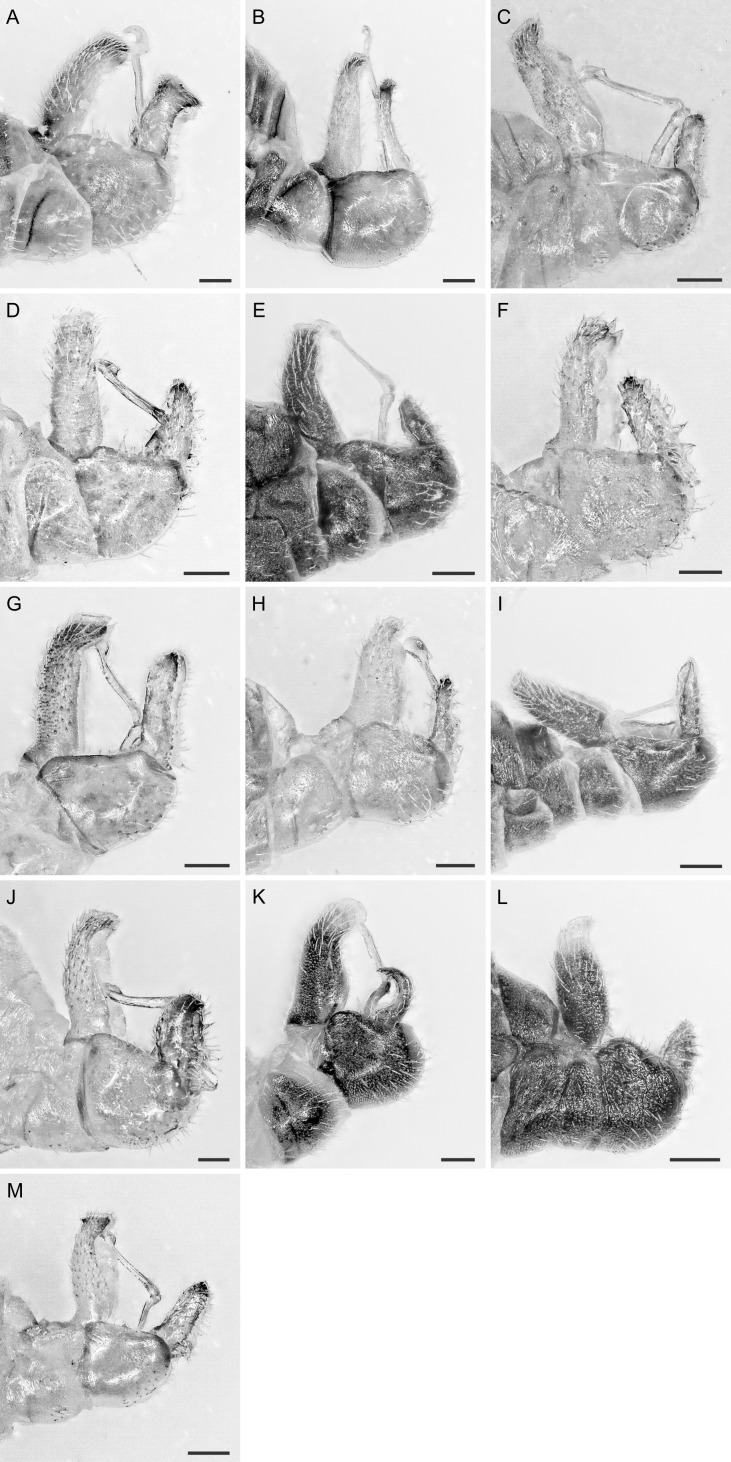
Overview of male terminalia of psyllid species in Luxembourg (Psyllidae I). **A** *Arytainagenistae* (Latreille, 1804) **B** *Arytainillaspartiophila* (Foerster, 1848) **C** *Cacopsyllaaffinis* (Löw, 1880) **D** *Cacopsyllaambigua* (Foerster, 1848) **E** *Cacopsyllacrataegi* (Schrank, 1801) **F** *Cacopsyllamali* (Schmidberger, 1836) **G** *Cacopsyllamelanoneura* (Foerster, 1848) **H** *Cacopsyllaperegrina* (Foerster, 1848) **I** Cacopsyllasp.gr.pruni (Scopoli, 1763) **J** *Cacopsyllapulchra* (Zetterstedt, 1838) **K** *Cacopsyllapyri* (Linnaeus, 1758) **L** *Cacopsyllapyricola* (Foerster, 1848) **M** *Cacopsyllapyrisuga* (Foerster, 1848).

**Figure 15.**
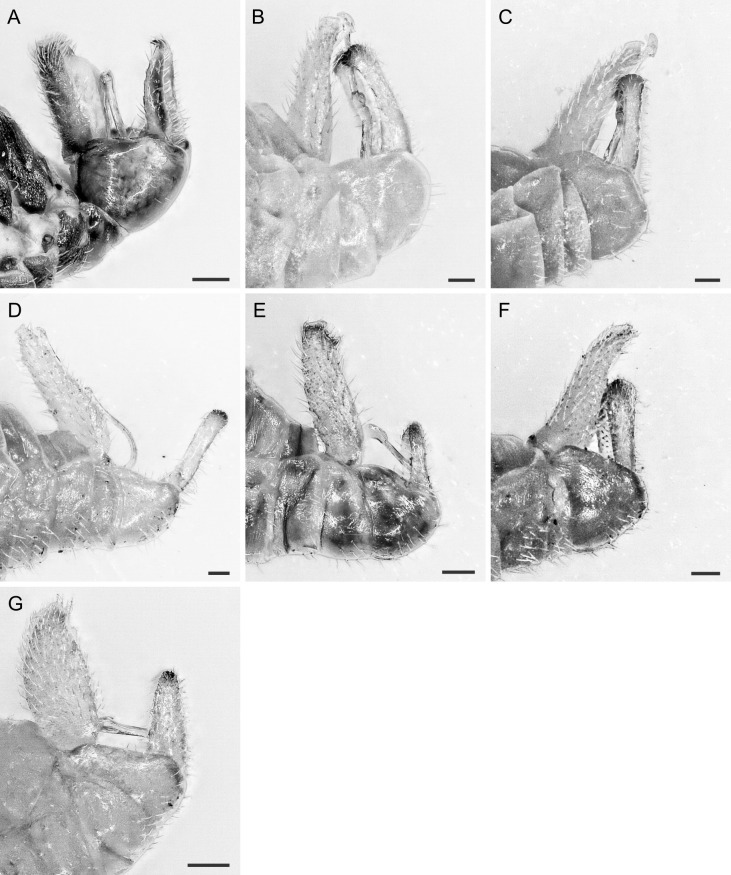
Overview of male terminalia of psyllid species in Luxembourg (Psyllidae II). **A** *Livillaulicis* Curtis, 1836 **B** *Psyllaalni* (Linnaeus, 1758) **C** *Psyllabetulae* (Linnaeus, 1758) **D** *Psyllafoersteri* Flor, 1861 **E** *Psyllahartigii* Flor, 1861 **F** *Spanioneurabuxi* (Linnaeus, 1758) **G** *Spanioneurafonscolombii* Foerster, 1848.

**Figure 16.**
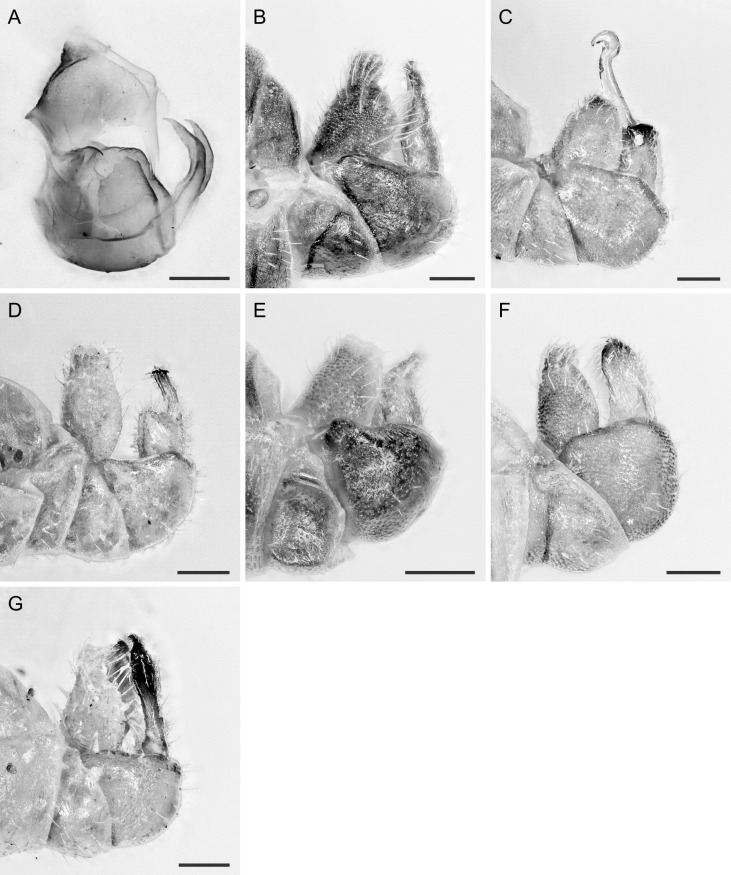
Overview of male terminalia of psyllid species in Luxembourg (Triozidae). **A** *Bactericerasubstriola* Ossiannilsson, 1992 **B** *Eryngiofagalautereri* Loginova, 1977 **C** *Triozaabdominalis* Flor, 1861 **D** *Triozacirsii* Löw, 1881 **E** *Triozagalii* Foerster, 1848 **F** *Triozaremota* Foerster, 1848 **G** *Triozaurticae* (Linnaeus, 1758).
